# Targeting GSK3 and Associated Signaling Pathways Involved in Cancer

**DOI:** 10.3390/cells9051110

**Published:** 2020-04-30

**Authors:** Przemysław Duda, Shaw M. Akula, Stephen L. Abrams, Linda S. Steelman, Alberto M. Martelli, Lucio Cocco, Stefano Ratti, Saverio Candido, Massimo Libra, Giuseppe Montalto, Melchiorre Cervello, Agnieszka Gizak, Dariusz Rakus, James A. McCubrey

**Affiliations:** 1 Department of Molecular Physiology and Neurobiology, University of Wrocław, Sienkiewicza 21, 50-335 Wroclaw, Poland; przemyslaw.duda@uwr.edu.pl (P.D.); agnieszka.gizak@uwr.edu.pl (A.G.); dariusz.rakus@uwr.edu.pl (D.R.); 2 Department of Microbiology and Immunology, Brody School of Medicine at East Carolina University, Greenville, NC 27858, USA; akulas@ecu.edu (S.M.A.); abramss@ecu.edu (S.L.A.); lssteelman@gmail.com (L.S.S.); 3 Dipartimento di Scienze Biomediche e Neuromotorie, Università di Bologna, 40126 Bologna, Italy; alberto.martelli@gmail.com (A.M.M.); lucio.cocco@unibo.it (L.C.); Stefano.ratti@unibo.it (S.R.); 4 Department of Biomedical and Biotechnological Sciences—Oncological, Clinical and General Pathology Section, University of Catania, 95123 Catania, Italy; saverio1979@hotmail.it (S.C.); mlibra@unict.it (M.L.); 5 Institute for Biomedical Research and Innovation, National Research Council (CNR), 90133 Palermo, Italy; giuseppe.montalto@unipa.it (G.M.); melchiorre.cervello@irib.cnr.it (M.C.); 6 Dipartimento di Promozione della Salute, Materno-Infantile, Medicina Interna e Specialistica di Eccellenza (PROMISE), University of Palermo, 90133 Palermo, Italy

**Keywords:** GSK-3, targeted therapy, natural products, drug resistance

## Abstract

Glycogen synthase kinase 3 (GSK-3) is a serine/threonine (S/T) protein kinase. Although GSK-3 originally was identified to have functions in regulation of glycogen synthase, it was subsequently determined to have roles in multiple normal biochemical processes as well as various disease conditions. GSK-3 is sometimes referred to as a moonlighting protein due to the multiple substrates and processes which it controls. Frequently, when GSK-3 phosphorylates proteins, they are targeted for degradation. GSK-3 is often considered a component of the PI3K/PTEN/AKT/GSK-3/mTORC1 pathway as GSK-3 is frequently phosphorylated by AKT which regulates its inactivation. AKT is often active in human cancer and hence, GSK-3 is often inactivated. Moreover, GSK-3 also interacts with WNT/β-catenin signaling and β-catenin and other proteins in this pathway are targets of GSK-3. GSK-3 can modify NF-κB activity which is often expressed at high levels in cancer cells. Multiple pharmaceutical companies developed small molecule inhibitors to suppress GSK-3 activity. In addition, various natural products will modify GSK-3 activity. This review will focus on the effects of small molecule inhibitors and natural products on GSK-3 activity and provide examples where these compounds were effective in suppressing cancer growth.

## 1. Introduction

Glycogen synthase kinase-3 (GSK-3) is a critical serine (S)/threonine (T) kinase which phosphorylates numerous proteins [1]. These phosphorylation events often result in the inactivation of the proteins by targeting them for proteasomal degradation. However, sometimes after phosphorylation the protein will have an altered subcellular location which can confer different biochemical activities. The number of GSK-3 substrates is very high and was summarized in many recent review articles [2,3,4]. It is perhaps due to the large number of substrates that GSK-3 can have different roles in either promoting or repressing cellular proliferation. With regards to cancer, GSK-3 can focus as a tumor promoter or in some cases a tumor suppressor. We will discuss various situations where GSK-3 is functioning as a tumor promoter or a tumor suppressor.

GSK-3 is considered in some cases to lie in the crossroads of various biochemical pathways. One of the most common pathways often implicated in cancer is the EGFR/RAS/PI3K/PTEN/AKT/GSK-3/mTORC1 pathway. This pathway plays critical roles in normal cell growth as well as can be hyperactivated due to mutations at *RAS*, PI3K (*PIK3CA*), *PTEN,* and other component genes to various extents [5,6,7,8].

For example, the epidermal growth factor receptor (*EGFR*) and other growth factor receptor genes are mutated or overexpressed in various cancers, the *KRAS* gene is often deregulated (close to 95%) mutated in pancreatic cancers, the *PIK3CA* (PI3K) gene is frequently disrupted in certain types of breast cancer (hormone-responsive breast cancers), and the *PTEN* gene, a tumor suppressor protein is mutated in various cancers. When these genes are mutated or aberrantly expressed, AKT becomes activated. AKT is also a S/T kinase and one of its numerous targets is GSK-3. When GSK-3 is phosphorylated by AKT, GSK-3 becomes inactivated and targeted for proteasomal degradation [9,10]. Other kinases such as mitogen-activated protein kinase (MAPK, ERK1/2) can phosphorylate and inactivate GSK-3β [11]. The presence of inactive or lower amounts of active GSK-3 has multiple consequences. When TSC2 and mTOR are not phosphorylated and inactivated by GSK-3, the mTORC1 complex is active and can result in the translation of various growth regulatory mRNAs and proliferation occurs.

GSK-3 can regulate NF-κB activity. GSK-3 can phosphorylate S8, S17, S31 and S43 of the NF-κB essential modifier (NEMO) which results in its stabilization. NEMO interacts with IκB kinases (IKK) and is essential for NF-κB activity [12]. Point mutations in NEMO at S8, S17, S31 and S43 result in its destabilization, proteasomal degradation and thus, reduced NF-κB activity. A consequence of inactive GSK-3 is that there is decreased NF-κB activity and NF-κB cannot induce the transcription of various genes involved in inflammation and metastasis which are often aberrantly regulated in cancer [13,14]. Thus, the cancer cells may not proliferate and invade in the absence of GSK-3 and NF-κB activity. Overexpression of GSK-3β can also result in BCLXL expression and resistance to tumor necrosis factor-related apoptosis-inducing ligand (TRAIL)-mediated apoptosis [15].

**Figure 1:**
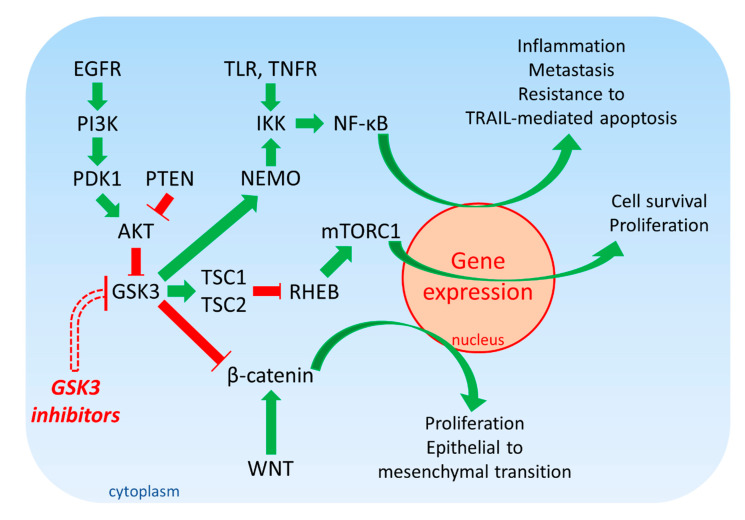
**Overview of EGFR/PI3K/PDK1/AKT/GSK-3/mTORC1 Signaling.** Green arrows indicate stimulation, blocked red arrows indicate inhibition.

An additional pathway that is regulated by GSK-3 is WNT/β-catenin. This pathway is also important in proliferation as well as the epithelial to mesenchymal transition (EMT) which is critical for cancer metastasis. When active, GSK-3 can phosphorylate β-catenin on three residues which results in its proteasomal degradation and many genes important in cell proliferation are not transcribed. Mutations at three residues on β-catenin prevent GSK-3 from phosphorylating them and thus, β-catenin is not able stimulate gene transcription and promote EMT [16,17]. An introductory diagram of the effects of GSK-3 on the EGFR/RAS/PI3K/PTEN/AKT/GSK-3/mTORC1 and NF-κB and WNT/β-catenin pathways is presented in Figure 1.

In addition, GSK-3β phosphorylates other key proteins in the WNT/β-catenin complex (e.g., adenomatous polyposis coli [APC], AXIN, low-density lipoprotein receptor-related protein 5/6 [LPR5/6]). This complex is involved in EMT which is critical for cancerous as well as normal growth. The roles of GSK-3β in cancer may differ according to cancer type and genetic mutations. AXIN may also have mutations in the GSK-3β phosphorylation sites which can alter its ability to be phosphorylated and inactivated. If β-catenin activity is increased due to the inability of GSK-3β to phosphorylate it and inactivate it, increased proliferation and drug resistance may occur. Additional studies showed that GSK-3β may exert also positive effects on cell growth. 

### 1.1. The GSK-3 Family Consists of GSK-3α and GSK-3β 

The *GSK3* gene family consists of two highly related genes, *GSK3A* and *GSK3B*. *GSK3A* encodes a 51 kDa protein and *GSK3B* encodes a protein of 47 kDa [1,3,4]. The two GSK-3 isoforms have 84% overall identity. The GSK-3α and GSK-3β have 98% identity in their catalytic domains; however, they diverge in their unique N- and C-terminals. The two GSK-3 isoforms have a bi-lobular structure, consisting of a large C-terminal globular domain which contains the catalytic domain, and a small N-terminal which contains the ATP binding site.

The two GSK-3 isoforms have distinctive functions and are not redundant. There are some differences in the expression of GSK-3α and GSK-3β. In some cells, the GSK-3α is the isoform that regulates NF-κB and cAMP response element in the response of bovine endothelial cells to *Staphylococcus aureus* infection [18]. GSK-3α and GSK-3β were also shown to have different roles in regulation of interleukin-12p40 expression in endothelial cells treated with *Staphylococcus aureus* peptidoglycan [19]. The phenotypes of *GSK3A* and *GSK3B* gene knockout (KO) mice are different. *GSK3A* knockout mice develop but they have brain problems and male infertility due to defects in sperm motility [20].

In conditional mouse knockout studies, deletion of GSK-3β expression was postulated to be necessary for the establishment of myelodysplastic disease syndrome (MDS) while both GSK-3β and GSK-3α were shown to be necessary for progression to acute myeloid leukemia (AML) [21]. 

There are also differences in the roles of GSK-3 isoforms in Alzheimer’s disease [22,23]. However, more research needs to be performed in this area to elucidate the different roles of GSK-3α and GSK-3β in various physiological processes.

### 1.2. Expression of the GSK-3 Isoforms in Different Human Tissues 

The expression of the two GSK-3 isoforms was examined in 27 different tissue types from 95 humans (normal) by Human protein analysis (HPA) and RNA-seq analysis (Table 1) [24]. The authors of this study created a new version of the Human Protein Atlas in 2014. This data base integrates RNA and protein expression data corresponding to ∼80% of the human protein-coding genes. Table 1 illustrates that GSK-3α is expressed at higher levels than GSK-3β. Moreover, certain tissues such as the brain have higher levels of GSK-3 expression than other tissues such as pancreas.

### 1.3. Biochemical Roles of GSK-3 in Cancer 

**Figure 2:**
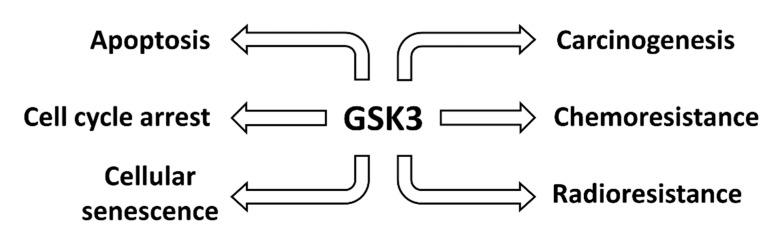
**Effects of GSK-3 on Various Cancers**. Aberrant GSK-3 expression can influence the development and progression of human cancer.

GSK-3 expression can affect multiple biochemical processes involved in cancer. A brief overview of some of the roles of GSK-3 in cancer is presented in Figure 2.

### 1.4. Examples of Studies Documenting the Roles GSK-3 Isoforms in Human Cancer

Many studies showed that GSK-3 can have different roles in human cancer. Table S1 presents a listing of some of the studies which illustrate that GSK-3 can have tumor suppressor or tumor promoter roles in human cancer. This table is organized in an alphabetical fashion and where possible, we have included studies which suggest that GSK-3 can have tumor promoter or tumor suppressor roles in the same tumor type. This issue complicates treatment with GSK-3 inhibitors unless the precise signaling pathway that is aberrant is known, as treatment with GSK-3 inhibitor could inhibit or in some cases enhance tumor growth.

## 2. GSK-3 Inhibitors

Numerous GSK-3 inhibitors were developed by multiple pharmaceutical companies since the initial characterization of the biochemical effects of GSK-3 and the association of GSK-3 with many common human disease states [23,25,26,27]. One of the reasons for the development of GSK-3 inhibitors is that GSK-3 is a regulator of NF-κB which is often aberrantly expressed in cancer and immunological disorders and has many targets which are pro-inflammatory genes which drive progression [28]. In this review, we will focus on novel GSK-3 inhibitors as well as novel combinations of GSK-3 inhibitors and chemotherapeutic approaches. A list of GSK-3 inhibitors and other agents which may inhibit GSK-3 activity that were examined in pre-clinical studies is presented in Table S2.

Lithium is a well-known GSK-3 inhibitor which was initially and still is used for the treatment of various neurological disorders including bipolar disorder (manic depression). It can inhibit proliferation of the human esophageal cancer cell line Eca-109 as well as other cell lines of different tissue types by inducing the phosphorylation of GSK-3β at S9 which can lead to inactivation of GSK-3. This in turn led to the stabilization of free β-catenin in the cytoplasm. Lithium treatment resulted in a G_2_/M block in the cell cycle of the Eca-109 cells. Thus, in these studies, GSK-3 and GSK-3 was normally serving as a tumor promoter in these cells [29].

GSK-3 activity is required for the growth of certain mutant *KRAS*-driven tumors including pancreatic cancers such as MIA-PaCa-2 cells. However, GSK-3 activity is not required for the growth of mutant *KRAS*-independent tumors and cells such as A549 lung cancer cells. Inhibition of GSK-3 activity with GSK-3 inhibitors (Tideglusib, AZD1080, and BIO) altered phosphorylation of numerous of GSK-3 substrates including: T53 on c-MYC, and S33/S37/T41 on β-catenin. GSK-3 blockade was determined to inhibit the proliferation of primary pancreatic cancer cells with *KRAS* mutations at G12D, G12V or G12C in mouse xenograft models as well as metastatic patient-derived xenograft (PDX) models made with PDAC cells isolated from patients who had progressed after chemo- or radiation therapies. In this scenario, inhibition of GSK-3 resulted in increased β-catenin and c-MYC activities which abrogated *KRAS*-dependent tumor growth [30].

The effects of the AR-A014418 GSK-3 inhibitor were examined on three pancreatic cancer cell lines. Treatment of the pancreatic cancer cell lines with AR-A014418 resulted in inhibition of growth and apoptosis. Reduced GSK-3 phosphorylation resulted in decreased activity of NOTCH pathways components. Ectopic expression of active NOTCH1 prevented the ability of AR-A014418 to inhibit growth. NOTCH1 was demonstrated to bind GSK-3α. In this scenario, the GSK-3 inhibitor was inducing the de-stabilization and lowering the level of NOTCH1. Thus, in this cancer type, GSK-3 was functioning as a tumor promoter by stabilizing NOTCH1 [31].

**Figure 3:**
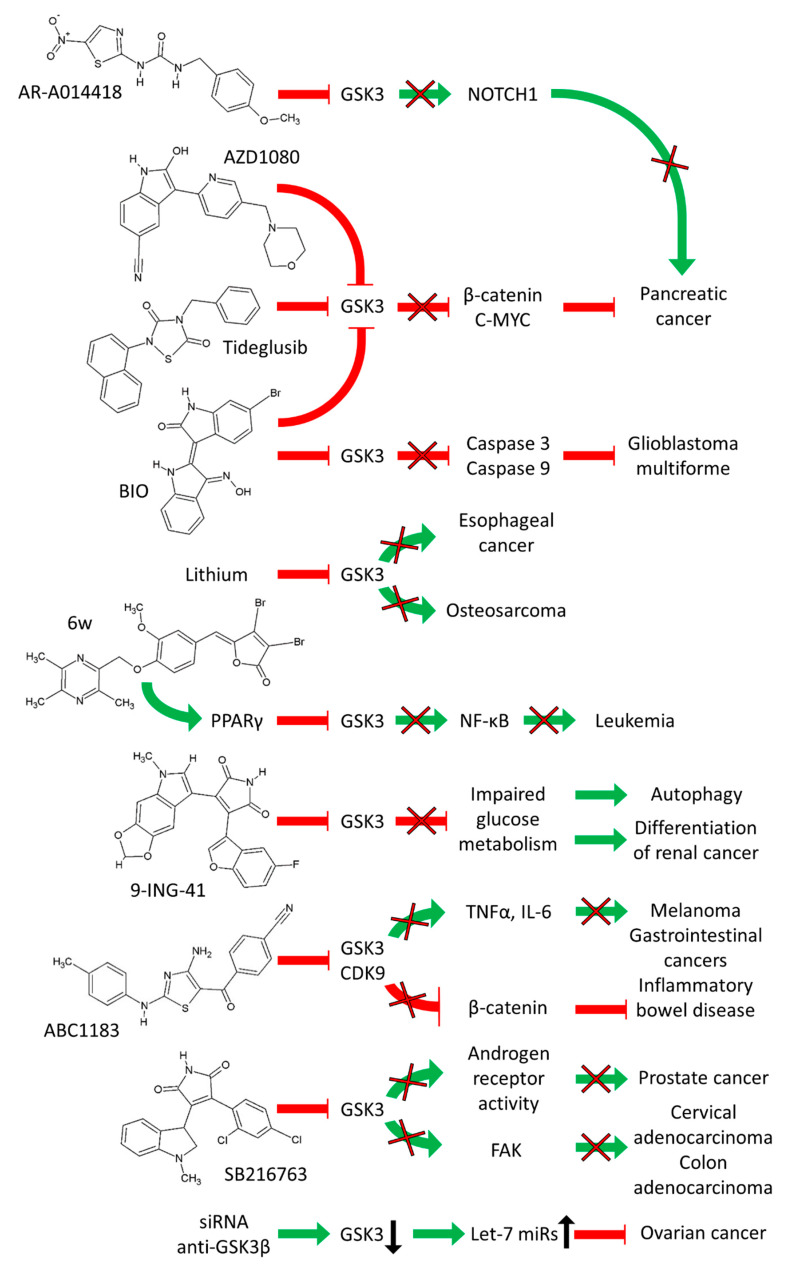
**Effects of GSK-3 Inhibitors on Various Cancers.** Green arrows indicate stimulation, blocked red arrows indicate inhibition.

A diagram of the effects of GSK-3 inhibitors on various cancers is presented in Figure 3.

Suppression of GSK-3 activity was shown to induce differentiation of renal cancer cells. This occurs, in part, by impaired glucose metabolism. This was determined by both pharmacological inhibition with the 9-ING-41 GSK-3 inhibitor as well as siRNA knockdown of GSK-3 in two renal cancer cell lines. Cell cycle arrest at G_0_/G_1_ and G_2_M phases was observed after treatment with the GSK-3 inhibitor. Suppression of GSK-3 in the renal cancer cells resulted in a differentiated phenotype as well as the induction of autophagy which was most likely due to impaired glucose metabolism which resulted in altered energy balance. The effects of the GSK-3 inhibitor were also examined in tumor xenograft studies. In these studies, GSK-3 was normally serving to promote tumor growth and treatment with the 9-ING-41 GSK-3 inhibitor suppressed GSK-3 activity and resulted in the induction of cell cycle arrest, autophagy and differentiation [32]. Interestingly, 9-ING-41 is one of the few GSK-3 inhibitors which is in clinical trials with various types of cancer patients (See Section 4).

Glioblastoma multiforme (GBM) is a malignant and aggressive type of brain tumor. The ability of the GSK-3 inhibitor IX to suppress the growth of GBM cells was determined. The GSK-3 inhibitor IX (BIO) increased the expression of the caspase-3 and caspase-8 proteins and induced apoptosis of GBM cells. The GSK-3 inhibitor induced G_2_/M cell cycle arrest of GBM cells [31].

Seventeen new 3,5-diamino-N-substituted benzamide compounds were designed as novel GSK-3β inhibitors. The novel compounds were evaluated both in vitro and in vivo in tumor models with human colon cancer cells. Some of the compounds exhibited selectivity against GSK-3β [33].

RNA production is regulated in part by cyclin-dependent kinase 9 (CDK9) and the transcription elongation factor B. This complex can regulate the RNA expression of oncogenes and genes involved in inflammatory responses. ABC1183 is a novel diaminothiazole that inhibits GSK3α and β and CDK9. ABC1183 inhibits the growth of a numerous cancer cell lines by decreasing cell survival by inducing G2/M arrest and through altering GSK-3 and WNT/β-catenin signaling. ABC1183 was determined to suppress tumor growth in the absence of organ and hematopoietic toxicity. The expression of tumor necrosis factor alpha (TNF-α) and interleukin-6 (IL-6) were suppressed. These pro-inflammatory factor/cytokines have important roles in tumor development. Thus, in this scenario, GSK-3 was serving as a tumor promoter and its inhibition suppressed WNT-β-catenin expression. GSK-3 under some circumstances can promote WNT-β-catenin stability. ABC1183 had anti-tumor effects against multiple tumor types including melanoma and gastrointestinal tumors. ABC1183 also possessed anti-inflammatory effects against inflammatory bowel disease (IBM) [34].

High levels of c-JUN are detectable in invasive breast cancer cells as well as in aggressive breast tumors. This resulted from prolonged c-JUN protein stability which was due in part to poor poly-ubiquitination of c-JUN. The constitutive photomorphogenesis protein 1 (COP1) is an E3 ligase which was determined to be responsible for c-JUN degradation in less-invasive breast cancer cells. An inverse relationship was observed between COP1 and c-JUN in a panel of breast cancer cell lines. However, COP1 was not the only molecule required to decrease c-JUN levels in invasive breast cancer cells, GSK-3β was also required to induce efficient c-JUN protein degradation. GSK-3 inhibitors (lithium chloride and SB21673) increased c-JUN protein levels in less-invasive breast cancer cells. Transfection of cells with vectors encoding COP1 and constitutively activated GSK-3β resulted in decreased levels of c-JUN expression and the invasive breast cancer cells. Thus, COP1 and GSK-3β interact to regulate c-JUN protein stability. This also resulted in the suppression of growth/migration in vitro and invasion of the breast cancer cells and in vivo. These results suggested that both COP1 and GSK-3β were acting to suppress breast cancer tumor growth and invasion and at least part of the effects was mediated by regulating the levels of c-JUN protein levels by controlling the stability of the protein [35].

The androgen receptor (AR) can also be phosphorylated by GSK-3. The GSK-3 inhibitors maleimide SB216763 and the aminopyrazole GSK inhibitor XIII will suppress AR-transcriptional activity as well as AR expression in prostate cancer cells. The GSK-3 inhibitor SB216763 suppressed the nuclear translocation of a green fluorescent AR fusion protein in PC3 cells. In contrast, other GSK-3 inhibitors had different effects as AR-A014418 promoted rather than decreased AR expression/function. These results suggest that some, but not all, GSK-3 inhibitors may have potential usefulness in prostate cancer therapy. In this scenario, SB216763 was suppressing AR-mediated effects [36].

h-prune is a protein which binds GSK-3. h-prune was shown to localize to focal adhesions and thus has effects on cellular migration. Treatment of cells with GSK-3 inhibitors (SB216763, lithium chloride) or siRNAs specific for GSK-3 and h-prune decreased the mobility of the HeLa S3 human cervix adenocarcinoma cells. The interaction between GSK-3 and h-prune was determined to be dependent on GSK-3 kinase activity. Suppression of either h-prune or GSK-3 resulted in inhibition of the phosphorylation of focal adhesion kinase (FAK) and RAC activation. Suppression of GSK-3 activity by GSK-3 inhibitors prevented paxillin disassembly as well as activation of FAK and RAC in HeLa S3, SW486 human colon adenocarcinoma cells and C57MG mammary epithelial cells. h-prune was determined to be expressed at high levels in CRC and pancreatic cancer cells. It was speculated that h-prune may be involved/associated with tumor aggressiveness and invasion. Thus, in this scenario, GSK-3 was acting as a tumor promoter and suppressing GSK-3 activity with inhibitors could reduce tumor aggressiveness by suppression of both the disassembly of paxillin and activation of FAK and RAC [37].

Certain 5-benzylidene-3,4-dihalo-furan-2-one derivatives are peroxisome proliferator-activated receptor (PPARγ) agonists. The effects of the PPARγ agonists including 22 rosiglitazone analogues, 5-benzylidene-3,4-dihalo-furan-2-one derivatives were examined on the U937 human leukemia cell line in mouse xenograft studies. The PPARγ agonist, [(Z)-3,4-dibromo-5-(3-methoxy-4-((3,5,6-trimethylpyrazin-2-yl)methoxy)benzylidene)furan-2(5H)-one (6w)] was observed to elicit its antitumor effects by inhibiting the PPARγ-dependent expression of GSK-3β and NF-κB. Thus, certain PPARγ agonists may function by suppressing GSK-3β, which in this scenario, was eliciting tumor promoter effects [38].

GSK-3β activity can also be inhibited by genetic means such as introduction of kinase-dead or kinase-inactive *GSK3B* mutants into cells. In addition, there is the potential to knockout GSK3B by the genome editing CRISP-Cas technology. N-acetyltransferase 10 (NAT10) is a protein that was determined to be important in colorectal cancers. NAT10 is a nucleolar protein and its subcellular locations and effects on cellular mobility were determined by introduction of kinase-inactive and wild-type forms of *GSK3B*. NAT10 was localized predominately to the nucleoli in normal cells but was present mostly at the leading edge of the CRC cells. NAT10 immunohistochemical staining was determined to reflect the depth of invasion and the ability of the CRC cells to metastasize. These two properties were associated with a poor prognosis. The localization of NAT10 at the leading edges was determined to result from increased NAT10 protein stability and nuclear export and inhibition of GSK-3β activity was required. This change in NAT10 localization resulted in increased cell motility and alterations in cytoskeletal dynamics. Thus, in this scenario, GSK-3β was functioning as a tumor suppressor in CRC cells and altered NAT10 localization was associated with a poorer clinical prognosis. Inhibition of GSK-3β activity by genetic approaches increased CRC invasive properties [39].

Suppression of GSK-3β activity by GSK3β-specific siRNAs in ovarian cancer BG1 cells resulted in increased Let-7 levels and decreased BG1 survival. Let-7 miRs are frequently detected at lower levels in cancer cells and thought to function normally as tumor suppressors. TP53 was determined to be a downstream effector of the impact of GSK-3β on Let-7 levels. Thus, GSK-3 can regulate Let-7 expression [40]. In this scenario, GSK-3 could be considered to be a tumor promoter as inhibiting GSK-3 resulted in increased levels of Let-7 which suppresses tumor growth. This is an example of the complexities of GSK-3. In this scenario, suppressing GSK-3 with a GSK-3 inhibitor, would inhibit cancer growth, in this case by increasing the expression of a miR.

GSK-3 inhibitors were shown to effect certain properties of stem cells [41]. GSK-3 is essential in the development of mixed lineage leukemias (MLL) in part by phosphorylation of the cyclin-dependent kinase inhibitor p27^Kip1^. This was shown by treatment with the GSK-3 inhibitors GSK-3 IX (BIO) and SB216763. In this scenario, GSK-3 was functioning as a tumor promoter. Suppression of GSK-3 activity may prove effective in the therapy of certain leukemias [7,42].

β-catenin becomes activated during the development of MLL leukemia stem cells (LSC). β-catenin was determined to be critical for the establishment of the MLL LSCs as well as their drug resistance. Suppression of β-catenin with GSK-3 inhibitors (lithium chloride, SB216763 and GSK-3 IX) resulted in reversion of MLL LSCs to a pre-LSC stage and reduced their growth. Conditional deletion of β-catenin eliminated the oncogenic potential of the MLL LSC. Established MLL LSCs were determined to be resistant to GSK-3 inhibitors. The MLL LSCs could be rendered sensitive to GSK-3 inhibitors by suppression of β-catenin. Some of the effects of the GSK-3 inhibitors were due to their prevention of p27^Kip1^ phosphorylation by GSK-3. In this scenario, GSK-3 was playing a critical role in the generation and drug resistance of MLL LSC and suppression of GSK-3 activity by GSK-3 inhibitors inhibited the development of MLL LSC [43].

GS87 is a novel GSK-3 inhibitor that was isolated upon screening for more effective inhibitors that induce AML differentiation. GS87 inhibits both GSK-3α and GSK-3β with IC_50_s of approximately 415 and 521 nM respectively, as determined by using radioactive in vitro kinase assays. Kinase profiling revealed that GS87 was highly specific for GSK-3 and able to induce AML differentiation more effectively than other GSK-3 inhibitors. GSK-3 was acting as a tumor promoter. GS87 had little effects on normal bone marrow cells [44].

Thiadiazolidinone (TDZD) is a non-competitive inhibitor of GSK-3. Treatment of human myeloma cells with TDZD resulted in Forkhead transcription factor (FOXO3a) activation. TDZD induced apoptosis in primary myeloma cells but not in normal CD34 cells or primary hematopoietic cells. GSK-3 was acting as a tumor promoter [45].

### 2.1. Combining GSK-3 Inhibitors with Chemotherapeutics, Radiation or Autophagy Inhibitors

The effects of combining GSK-3 inhibitors with radiation and chemotherapy were examined. GSK-3 has also been implicated in DNA repair. GSK-3β can have effects on DNA repair through phosphorylation of DNA repair factors which influence their ability to bind chromatin. GSK-3β can play roles in both base excision repair as well as double strand break repair. Some of these processes are mediated by activation of NF-κB and inhibition of apoptosis. Moreover, certain GSK-3β inhibitors will increase the sensitivity of certain cancer cells to chemotherapy. In these scenarios, inhibition of GSK-3 was acting in a tumor suppressor role, thus, GSK-3, itself, was acting in a tumor promoter role [46].

**Figure 4:**
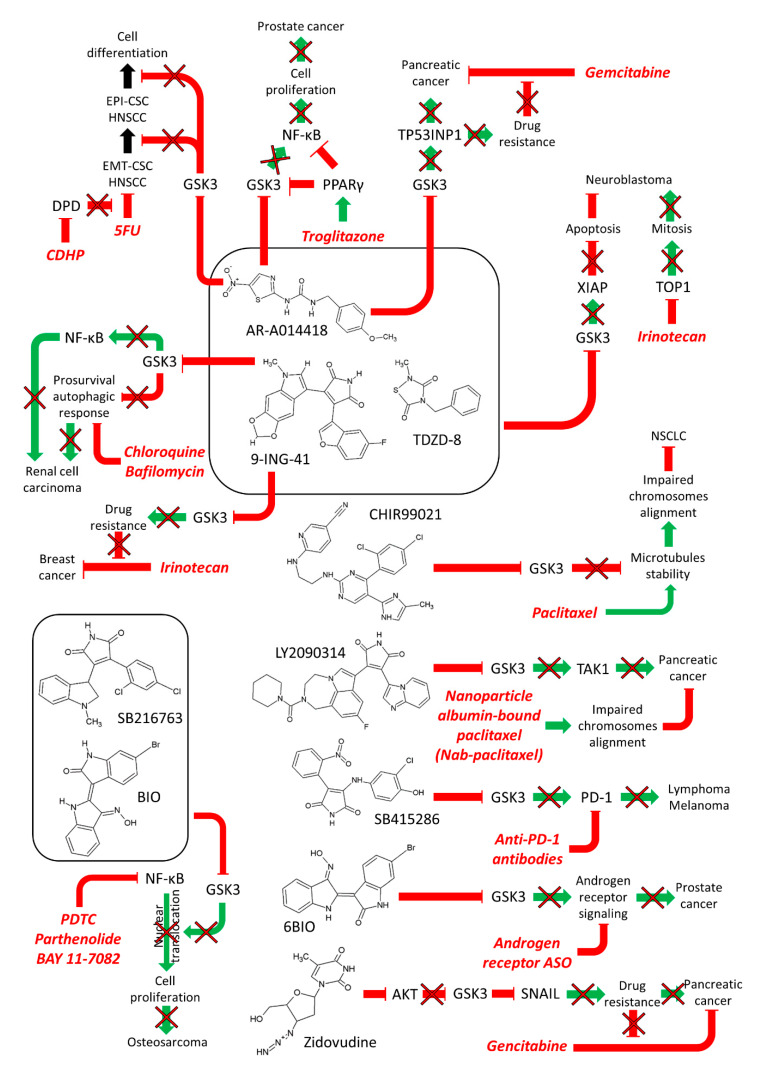
Effects of Combining GSK-3 Inhibitors with Chemotherapy or Immunotherapy. Green arrows indicate stimulation, blocked red arrows indicate inhibition.

Combining GSK-3 inhibitors with paclitaxel was observed to increase the death of non-small cell lung cancer cells (NSCLC). GSK-3 plays roles in the correct alignment of chromosomes on the metaphase plate through regulation of microtubule stability. GSK-3 is overexpressed and hyperactivated in certain NSCLC cells. CHIR99021 inhibits both GSK-3α and GSK-3β. CHIR99021, when combined with paclitaxel was observed to inhibit the proliferation NSCLC cell lines in a synergistic fashion. Some of synergistic effects were determined to be from a defect in chromosomal alignment. Combining the GSK-3 inhibitor CHIR99021 with paclitaxel increased the growth inhibition of NSCLC in both cell-based and tumor xenograft models [47]. A diagram of some of these combinations is presented in Figure 4.

GSK-3 plays important roles in the mitotic checkpoint. If chromosomes are unattached to spindle microtubules, the mitotic checkpoint is blocked, and proper chromosome segregation does not occur. The presence of kinetochores that are not attached results in the formation of the mitotic checkpoint complex (MCC) that is composed of multiple proteins. This results in the inhibition of the onset of anaphase. GSK-3 inhibitors such as SB415286, RO318220 and lithium chloride were observed to suppress spindle toxin-induced mitotic arrest in various cell lines including HeLa (cervical carcinoma), HCT116 (colon carcinoma) and HT1080 (fibrosarcoma). In addition, genetic approaches to suppress GSK-3 activity such as either knockout or RNAi reduced mitotic arrest mediated by paclitaxel. GSK-3 was determined to be required for localization of the MAD2, BUBR1 and BUB2 at the kinetochores and for assembly of the MCC in the spindle toxin treated cells. Inhibition of WNT and PI3K/AKT signaling pathways induced a longer mitotic arrest in the present of paclitaxel than that observed in the absence of the inhibitors. These studies illustrate a role for GSK-3 in spindle toxin-induced mitotic arrest and in this case, GSK-3 inhibitors may promote cellular proliferation as they suppressed mitotic arrest [48].

GSK-3 can stabilize transforming growth factor beta-activated kinase 1 [(TAK1) a.k.a mitogen-activated protein kinase kinase kinase 7 (MAP3K7)] which plays a key role in pancreatic cancer. Suppression of GSK-3 also resulted in reduction of TAK1 levels. Studies by this group indicated that TAK1 was affecting HIPPO and ubiquitination pathways. The GSK-3 inhibitor LY2090314 suppressed TAK1 levels. LY2090314 plus nab-paclitaxel combined treatment increased the survival of mice in orthotopic pancreatic tumor models. In this pancreatic cancer model, GSK-3 was normally acting as a tumor promoter by affecting the activity of TAK1 [49].

Neuroblastoma is a very aggressive disease in pediatric patients. The effects of various GSK-3 inhibitors (AR-A01441, TDZD-8, and 9-ING-41) on the growth of neuroblastoma cells were determined. 9-ING-41, is a clinically relevant GSK-3 inhibitor. 9-ING-41 suppressed the growth of neuroblastoma cells more effectively than the other GSK-3 inhibitors. 9-ING-41 suppressed the expression of the anti-apoptotic molecule X-linked inhibitor of apoptosis protein (XIAP). Combining 9-ING-41 with clinically relevant doses of the topoisomerase inhibitor Camptosar (a.k.a. CPT-11, irinotecan) led to enhanced anti-tumor effects in mouse xenograft models [50].

The effects of 9-ING-41 were examined either by itself or in combination with autophagy inhibitors such as chloroquine and bafilomycin on renal cancer cell (RCC) lines and in cells from patients with advanced RCC. By itself, 9-ING-41 induced apoptosis and cell cycle arrest. In combination with autophagy inhibitors, the effects increased. 9-ING-41 increased the anti-tumoral effects of certain targeted therapeutics and was observed to potentiate the cytotoxic effects of cytokine-activated immune cells on RCC. Thus, in this scenario, inhibiting GSK-3 activity with 9-ING-41 prevented RCC growth and 9-ING-41 synergized with autophagy inhibitors [51].

### 2.2. Combining GSK-3 Inhibitors and Immunotherapy

Immune checkpoint blockade (ICB) has become an increasingly popular approach in cancer immunotherapy. However, many cancers remain insensitive to ICB and approaches to make these cancers sensitive are being investigated. GSK-3 was shown to be a regulator of PD-1 in T cells. GSK-3 inhibitors were determined to down-regulate PD-1 expression [52].

GSK-3 inhibitors such as GSK-3i and SB415286, suppress transcription of PD-1 expression in CD8+ T cells. Suppression of GSK-3 was as effective as αPD-1 and αPD-L1 blocking antibodies in preventing the growth of the B16 melanoma or the EL4 lymphoma in in vivo tumor studies. Conditional GSK-3 α and β knock-out mice also displayed reduced PD-1 expression and suppressed B16 primary metastasis to the same extent as PD-1 gene deficiency. These studies point to a central role of GSK-3 in controlling the expression of PD-1 and in this scenario GSK-3 was serving a tumor promoter role [53].

Treatment of CD8+ cytotoxic T lymphocytes with the GSK-3 inhibitor SB415286 resulted in increased transcription of TBX21 (T-bet) and decreased expression of PD-1 in both in vitro and animal studies. This led to increased cytotoxicity in both in vitro with EL4-OVA targets and OT-1 CD8+ CTLs, and animal studies with Murid herpes virus (MHN-68) and LCMV-CI13 virally infected cells. In the animal studies, enhanced viral clearance was observed upon treatment with the GSK-3 inhibitor [54].

GSK-3 inhibitors have also been evaluated in the suppression of PD-1 expression with antigen-specific chimeric antigen receptor T (CAR-T) cells. SB216763 GSK-3 inhibitor treatment of GBM-specific CAR-T cells resulted in reduced FasL and PD-1 expression, and increased T cell proliferation. This led to the development of CAR-T effector memory phenotype. In GMB-bearing mice, treatment with the GSK3-suppressed CAR-T cells led to 100% tumor elimination upon tumor-re-challenge experiments. In addition, the presence of memory CAR-T cells in secondary lymphoid organs increased [55].

Treatment of NK cells with GSK-3 inhibitors LY2090314, tideglusib or SB415286, increased NK activity in AML-NK cells. The increased activity of the NK cells was shown to be due to increased tumor necrosis factor-α (TNF-α) levels [56].

The ability of GSK-3 inhibitors to sensitize human gastric adenocarcinoma AGS cells to TNF-related apoptosis-inducing ligand (TRAIL) was examined. Many tumor cells develop resistance to treatment with TRAIL as a single agent. Two GSK-3 inhibitors, SB415286 and LiCl, were shown to sensitize established gastric adenocarcinoma cells to TRAIL-mediated apoptosis, but they did not sensitize “normal” primary gastric epithelial cells to TRAIL.

The sensitization was mediated in part by increasing the levels of caspase 8. Suppressing wild-type (WT)-TP53 activity by introduction of siRNA specific for WT-TP53 depressed the ability of GSK-3 inhibitors to induce sensitization to TRAIL. In this scenario, GSK-3 inhibitors were acting in a TP53-dependent fashion to induce sensitivity to TRAIL. Thus, GSK-3 was promoting resistance to TRAIL and acting as a tumor promoter [57].

Enzastaurin was developed as a PKC-β inhibitor. One of its targets is GSK-3. It was evaluated in clinical studies with various advanced or metastatic cancer patients, often in combination with bevacizumab which inhibits the vascular endothelial growth factor A (VEGFa). Encouraging results were observed with patients with ovarian cancers. 50.4 % of ovarian cancer patients remained without disease progression after 6 months [58]. A phase II clinical trial was performed with enzataurin and bevacizumab in patients with glioma. Early response was associated with longer progression free survival. The combined treatment was well tolerated, and survival times were similar to that observed in patients treated with bevacizumab [59].

### 2.3. Combining GSK-3 Inhibitors with Other Inhibitors or Agonists

Inhibition of GSK-3 activity with lithium chloride, SB216763, inhibitor IX (BIO) or with siRNA specific for GSK-3β resulted in suppression of GSK-3β and NF-κB activities and cellular proliferation and induction of apoptosis in human osteosarcoma cells in vitro and in mouse xenograft studies. Combining GSK-3 and various NF-κB inhibitors, such as ammonium pyrrolidine dithiocarbamate (PDTC), parthenolide, or BAY 11-7082, with certain chemotherapeutic drugs increased cell death both in vitro and in mouse xenograft studies. Human osteosarcoma patients who had higher levels of active GSK-3β and nuclear-localized NF-κB had a worse prognosis than those patients with lower levels of active GSK-3 and NF-κB in terms of patient survival periods. In this scenario, suppression of GSK-3 was inhibiting tumor growth and GSK-3 activity was normally serving as a tumor promoter by stimulating NF-κB activity [60].

Certain PPARγ ligands will reduce the proliferation of human prostate cancer cells. Previous studies showed that GSK-3β and NF-κB have important roles in prostate cancer. Troglitazone is a PPARγ agonist. It has anti-diabetic and anti-inflammatory properties and was used to treat diabetes patients. However, since 2000 troglitazone has been removed from the markets because of hepatotoxicity. Troglitazone induced the expression of PPARγ in the nucleus of PC3 but not LNCaP prostate cancer cells. GSK-3β expression and NF-κB activities have important roles in prostate cancer development. Troglitazone suppressed cell growth, G_0_/G_1_ block and apoptosis to similar extents in both PC3 and LNCaP prostate cancer cells. Troglitazone treatment inhibited GSK-3β expression as well as activation of NF-κB. Interestingly, treatment of prostate cancer cells with troglitazone and either a GSK-3β inhibitor (AR-A014418) or GSK-3β siRNA potentiated the effects of troglitazone on suppression of NF-κB activity. This increased apoptosis and cell death. In this scenario, inhibiting GSK-3 was suppressing NF-κB activation which resulted in cell death of prostate cancer cells [61]. Novel troglitazone derivatives are being designed and some show anti-proliferative effects on certain cancer types [62].

### 2.4. Effects of GSK-3 Inhibitors on Drug Resistance in Cancer Cells

GSK-β plays roles in the sensitivity to chemotherapeutic drugs, signal transduction inhibitors, nutraceuticals and other small molecule inhibitors [63]. A diagram summarizing some of the effects of combining GSK-3 inhibitors with other drugs and inhibitors is presented in Figure 4**.**

We will consider some of the newer reports on the effect of GSK-3 inhibitors on drug resistance in cancer cells. 6-bromo-indirubin-3’oxime (6BIO) is a GSK-3 inhibitor that was shown to improve the targeting of an antisense oligonucleotide (ASO) inhibitor in prostate cancer cells. 6BIO treatment resulted in inhibition of GSK-3α and GSK-3β and enhanced ASO function [64]. 6BIO reduced both androgen receptor (AR) and AR signaling in prostate cancer cells. 6BIO decreased the drug resistance of the AR splice variant AR-V7 which is important in prostate cancer drug resistance and cancer progression. When 6BIO was combined with AR-ASO, increased inhibition of AR signaling was observed. In this scenario, GSK-3α and GSK-3β were acting as tumor promoters and their suppression eliminated the growth of drug-resistant prostate cancer cells.

Head and neck squamous cells carcinomas (HNSCCs) are comprised by at least two types of cancer stem cells (CSCs). One HNSCC CSC phenotype expresses high levels of CD44 and epithelial-specific antigen (ESA) and has epithelial characteristics (Epi-CSC). The other HNSCC CSC phenotype has high CD44 and low ESA expression but has undergone EMT. This latter HNSCC CSC phenotype has migratory and mesenchymal properties (EMT-CSC). Both phenotypes are resistant to various apoptosis-inducing drugs such as 5-flurouracil (5FU). GSK-3β was shown to be involved in the self-renewal and switching of both types of HNSCC CSC phenotypes [65]. The CD44 high/ESA(low) HNSCC CSC group of cells had resistance to 5FU and expressed high levels of dihydropyrimidine dehydrogenase (DPD). 5-chloro-2,4-dihydroxpyridine (CDHP) suppresses DPD. Combining 5FU and CDHP resulted in enhanced apoptosis in CD44 (high)/ESA (low). Addition of a GSK-3β inhibitor AR-A014418 induced CD44 (high)/ESA (low) cells to undergo mesenchymal-to-epithelial transition (MET) back to CD44 (high)/ESA (high) cells. Furthermore, this combined treatment induced the pre-existing CD44 (high)/ESA (high) cells to differentiate. In summary, the combined treatment with CDHP and GSK-3β inhibitors stimulated 5FU-induced apoptosis of CD44 (high)/ESA (low) cells. These studies point to the usefulness of combining GSK-3 inhibitors with other classical chemotherapeutic drugs and other inhibitors (e.g., CDHP) in eliminating the CSC properties of certain cancers [66].

The effects of two GSK-3 inhibitors (9-ING-41 and 9-ING-87) were examined on breast cancer cells in the presence and absence of irinotecan in vitro and in PDX. The PDX models were created with cells obtained from metastatic pleural effusions from breast cancer patients whose cancers had become progressive and chemo-refractory. 9-ING-41 increased the effects of the chemotherapeutic drug irinotecan. These results indicate that inhibiting GSK-3 may overcome the drug resistance of certain cancer types such as breast [67]. In this scenario, GSK-3 is serving as a drug resistance promoter.

PANC-1 pancreatic cancer cells have elevated levels of tyrosine (Y)-216-phosphorylated GSK-3β (active form of GSK-3β) than non-neoplastic HEK293 cells. AR-A014418 is an inhibitor which suppresses GSK-3β. AR-A014418 inhibited the proliferation of PANC-1 cells and xenografts. This GSK-3 inhibitor was observed to synergize with gemcitabine, a nucleoside analog which is commonly used to treat PDAC patients. A common problem observed with treatment of PDAC with gemcitabine is the development of drug resistance. Gemcitabine treatment of PANC-1 resulted in increased levels of certain genes including TP53-induced nuclear protein 1 (TP53INP1) which is a gene involved in DNA repair and cell death. In contrast, the expression of this gene decreased by inhibition of GSK-3β [68]. Thus, GSK-3β inhibition suppressed some of the genes induced by gemcitabine which may be involved in drug resistance. In this scenario, GSK-3β was serving as a tumor promoter as inhibition of its activity suppressed growth.

### 2.5. Other Inhibitors Which Also Influence GSK-3 Activity

Some small molecule inhibitors that were originally developed to target other proteins will also suppress GSK-3 either directly or by targeting an upstream protein which inhibits GSK-3. [69].

GDC-0941 is a PI3K inhibitor. Treatment of GBM cells with GDC-0941 was determined to sensitize the cells to radiotherapy and reduced their chemoresistance to temzolomide [70].

The potential of targeting AKT was examined by many investigators and pharmaceutical companies due to its importance in regulating cellular proliferation and apoptosis. AktX is an AKT inhibitor. The effects of AktX and lithium chloride were examined on brain cancer cells. AktX suppressed AKT and increased active GSK-3β expression and inhibited glioma cell proliferation. [71].

Zidovudine is an antiviral drug. Zidovudine treatment was shown to sensitize gemcitabine-resistant pancreatic cells. Zidovudine inhibited the AKT/GSK-3β/SNAIL pathway and made the cells sensitive to gemcitabine. In this scenario, GSK-3β was activated due to inhibition of AKT phosphorylation. GSK-3β could then phosphorylate and inactivate SNAIL. These studies point to an approach to treat gemcitabine-resistant cells by combining zidovudine and gemcitabine [72]. In this scenario, GSK-3 was acting as a tumor suppressor.

Doxazosin is an antihypertensive drug. Interestingly, it was observed to inhibit PI3K/AKT signaling in GBM. Doxazosin treatment resulted in up-regulation of active GSK-3β and TP53. Importantly, administration of doxazosin was associated with low neurotoxicity as it has low cytotoxicity on primary astrocytes and hippocampal organotypic cultures [73].

### 2.6. Small Molecule Inhibitors and miRrs Which Interact to Regulate GSK-3 Activity in Drug Resistance

**Figure 5:**
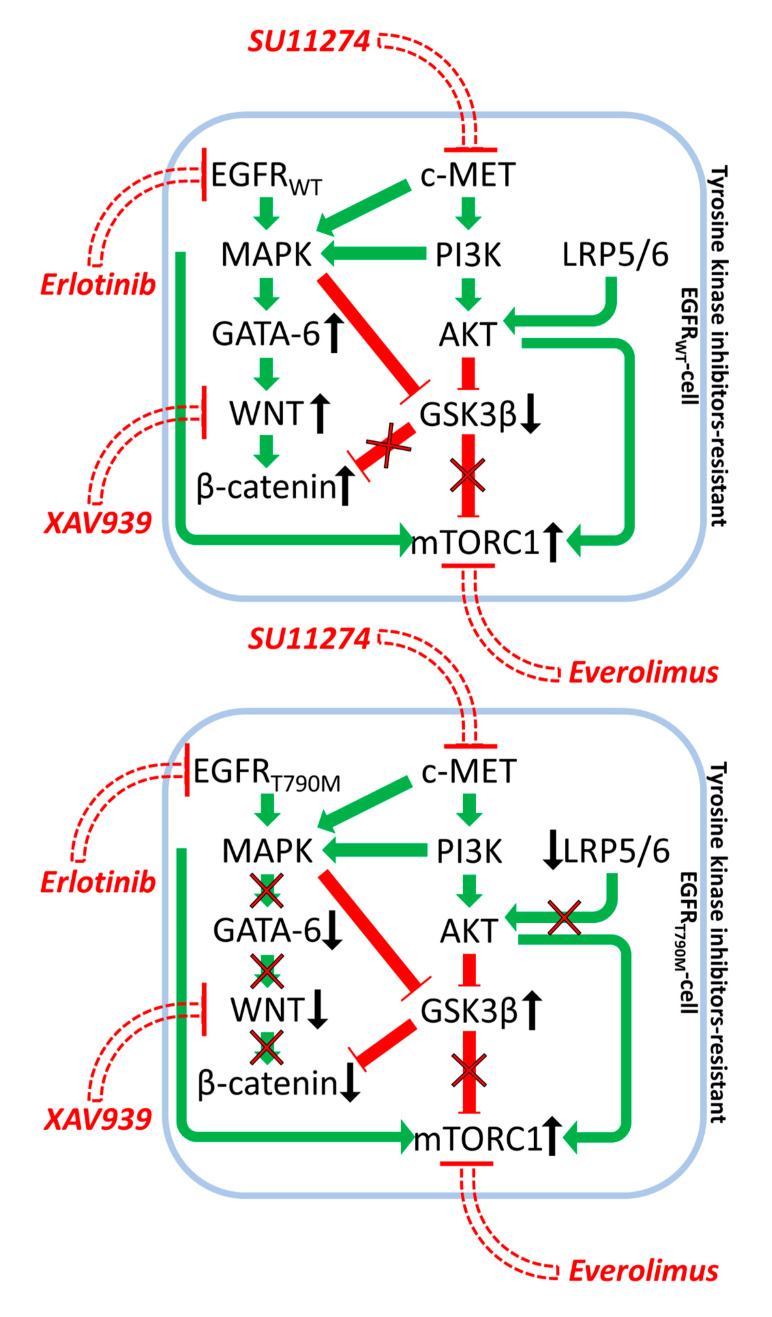
Effects of EGFR, mTORC1 and WNT Inhibitors on Growth Inhibition in Drug Resistance in Non-Small Cell Lung Carcinoma. Green arrows indicate stimulation, blocked red arrows indicate inhibition.

A mechanism by which NSCLC can become resistant to targeted therapeutics is by deregulated GSK-3 and β-catenin expression. Initially, depending on the genetic background of the patient, NSCLC cells are sensitive to the EGFR or c-MET inhibitors, but certain NSCLC are resistant to EGFR inhibitors such as erlotinib due to pre-existing EGFR mutations. The NSCLC H1975 and H2170 cells were investigated to determine how resistance to targeted therapeutics may develop. H1975 cells contain the T790M EGFR mutation while H2170 cells are WT at EGFR. These T790M EGFR mutation confers resistance to the EGFR inhibitor erlotinib. H2170 cells were made resistant to erlotinib and the c-MET inhibitor SU11274 by culturing the cells in increasing concentrations of the tyrosine kinase inhibitors (TKIs) for prolonged time periods. The WNT/β-catenin pathway was elevated in the TKI-resistant H2170 cells in comparison to TKI-sensitive H2170 cells. In addition, higher levels of phosphorylated, inactive GSK-3β were also detected in these cells. In H1975 cells, the levels of active β-catenin and GATA-6, and phosphorylated GSK-3β were all down-regulated. Treatment of H1975 cells with the WNT inhibitor XAV939 did not significantly inhibit growth. However, upon combined treatment with the WNT inhibitor, the mTORC1 blocker (everolimus) and erlotinib of H1975 cells, which are normally insensitive to erlotinib, synergistic growth inhibition was observed. These studies point to the possibility of targeting TKI-resistant NSCLC with WNT, mTORC and either EGFR or c-MET inhibitors [74]. In this scenario, GSK-3 was serving as a tumor promoter as active GSK-3 decreased in the drug-resistant cells and mTORC1 was elevated. A diagram depicting this model is present in Figure 5.

miRs can target mRNAs encoding GSK-3 and alter their stability. miR-101 was shown to suppress GSK-3β in glioblastoma. This resulted in restoration of the sensitivity of glioblastoma cells to temozolomide. [75].

## 3. Natural Products Which Modify GSK-3 Activity

The effects of curcumin, berberine and resveratrol on cancer and other diseases was recently summarized [76]. Curcumin can regulate the spleen tyrosine kinase (SYK) which is constitutively activated in certain B-lymphomas and required for their growth [77]. The effects of curcumin on SYK activity may be due to down regulation of AKT which results in inhibition of GSK-3 phosphorylation and GSK-3 negatively affects SYK activity.

Curcumin was shown to have effects on colon carcinogenesis. Part of the effect of curcumin on colon carcinogens may be due to induction of GSK-3 activity and suppression of WNT/β-catenin signaling [78].

Berberine prevented the phosphorylation of GSK-3 in melanoma cells treated with alpha melanocyte stimulating hormone (α-MSH). Berberine suppressed induction of microphthalmia-associated transcription factor (MITF) and tyrosinase activity which normally occurs after α-MSH treatment. In this scenario, berberine was serving to inhibit AKT activity which in turn resulted in GSK-3 activity and suppressed the induction of MITF and downstream molecules including melanin. Thus, both berberine and GSK-3 were serving as tumor suppressors [79].

Berberine has multiple effects on cells including DNA replication and induction of reactive oxygen species (ROS). Combining berberine with the dual EGFR and HER receptor inhibitor lapatinib was shown to decrease the lapatinib-resistance of breast cancer cells. Lapatinib by itself can activate c-MYC, Nrf2 and GSK-3 signaling pathways. Nrf2 is an important regulatory of antioxidant molecules which are essential for cellular repair due to various harmful insults. The combined treatment with berberine and lapatinib induced higher levels of ROS in the cells which increased GSK-3 activity and decreased c-MYC expression. This made the lapatinib-resistant breast cancer cells sensitive to lapatinib [80].

Resveratrol was also shown to have some anti-cancer properties which are mediated at least in part by GSK-3. In breast cancer cells, a natural analog of resveratrol [3,5,4’-trimethoxystilbene] was shown to increase GSK-3 activity which suppressed WNT/β-catenin signaling and decreased invasion and migration. In addition, the resveratrol analog suppressed SNAIL, SLUG and vimentin expression while increasing E-cadherin expression [81]. Thus, resveratrol was suppressing some aspects of EMT, possibly by increasing GSK-3 activity. In this scenario, resveratrol and GSK-3 were serving as tumor suppressors.

In many cancer model systems, resveratrol is believed to increase GSK-3 activity by decreasing the activity of PI3K/AKT signaling. This results to increase GSK-3 activity which suppresses the proliferation of the cancer cells. This was observed in colon cancer [82] endometrial cancer [83], leukemia [84], pancreatic cancer [85], prostate cancer [86,87,88] ovarian cancer [89,90].

Apocynin is a nutraceutical structurally related to vanillin. It is produced by several plants. The effects of apocynin and resveratrol on pancreatic cells were determined to be due to decreased levels of phosphorylated GSK-3β and ERK1/2 present in the nucleus [85].

**Figure 6:**
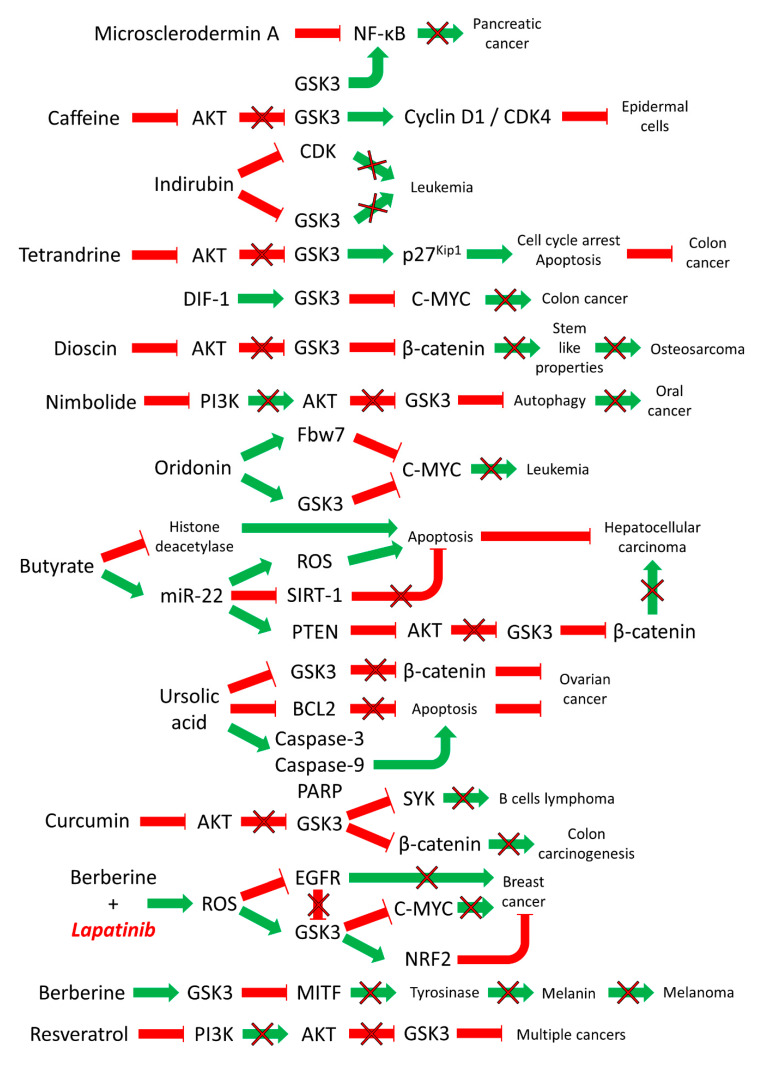
**Effects of Natural Products on GSK-3 Activity in Various Cancers**. Green arrows indicate stimulation, blocked red arrows indicate inhibition.

Microsclerodermin A is a marine-derived natural product. Microsclerodermin A inhibits NF-κB activity and induces apoptosis in certain cell types such as pancreatic cancer cells. NF-κB regulates the expression of hundreds of genes which often encode proteins involved in inflammation. Inflammation is a critical component of pancreatic cancer. NF-κB is constitutively activated in pancreatic cancer cells which is due in part to the presence of mutated *KRAS*. Microsclerodermin A inhibited NF-κB transcription which may be regulated by GSK-3β activity. In this scenario, GSK-3β was acting to regulate NF-κB activity which could be suppressed by microsclerodermin A [91]. A diagram depicting some of the effects of natural products on GSK-3 activity is presented in Figure 6.

Caffeine is present in coffee and tea. Interestingly, caffeine can also suppress the growth of certain cancers. Caffeine was determined to inhibit proliferation by inducing cycle arrest at the G_0_/G_1_ phase in JB6 mouse epidermal cells. Caffeine inhibited the phosphorylation of AKT and GSK-3β and GSK-3β was active. Caffeine suppressed the phosphorylation of the retinoblastoma protein as S780 and S807/S811 and inhibition of the cyclin D1-CDK4 complex [92].

Indirubin can be obtained the from the indigo plant. It is used in traditional Chinese medicine (TCM) to treat various diseases including leukemia. One of the targets of indirubin is GSK-3 and cyclin-dependent kinases (CDKs). It may function by competing with ATP binding to the ATP binding sites in the kinases [93].

Tetrandrine is a bis-benzylisoquinoline alkaloid. It can be isolated from herbs such as *Stephania tetrandra* S. Moore. It has various biological properties including a calcium channel blocker and anti-inflammatory and anti-cancer properties. Tetrandrine induced dephosphorylation and inactivation of AKT and activation of GSK-3 in HT-29 colon cancer cells. Tetrandrine induced up-regulation of the p27^Kip1^ inhibitors, arrest at the G_1_ phase of the cell cycle and apoptosis. Treatment of the cells with either GSK-3β inhibitors or siRNAs directed to GSK-3β could block the effects of tetrandrine. Transfection of cells with WT GSK-3β also led to G_1_ arrest, cleavage of poly ADP ribose polymerase (PARP) and apoptosis. In this scenario, GSK-3β was functioning as a tumor suppressor and could be activated by the natural product tetrandrine [94].

Differentiation-inducing factor-1 (DIF-1) is a factor that is isolated from *Dictyostelium discoideum*. It has some anti-cancer properties which may be mediated by GSK-3β and WNT/β-catenin [95]. DIF-1 was determined to activate GSK-3 expression which in turn altered the levels of c-MYC in HCT-116 colon cancer cells. DIF-1 suppressed c-MYC mRNA levels by inhibiting TCF binding sites in the promoter region of c-MYC. The GSK-3β inhibitors lithium chloride and SB216763 were observed to suppress the effects of DIF-1 on c-MYC. These results indicated that the effects of DIF-1-induced c-MYC levels were mediated via activation of GSK-3. Thus, DIF-1 was with two effects on c-MYC levels by inhibiting promoter activity and by inducing protein degradation mediated by GSK-3β. These combined effects on c-MYC expression inhibited proliferation. In this scenario, GSK-3 was acting as a tumor suppressor to inhibit c-MYC levels [96].

Certain natural products will suppress the growth of cancer cells by repressing AKT/GSK-3/β-catenin. They often act by repression of AKT expression which in turn inhibits the phosphorylation of downstream GSK-3β at S9. GSK-3β is active and can inhibit cell proliferation which inhibits tumor growth. A recent study examined the effects of dioscin on osteosarcoma cells. Dioscin is obtained from *Discorea nipponica* Makino. Dioscin was determined to inhibit cell proliferation and induce apoptosis in osteosarcoma cells. Dioscin inhibited the stem-cell like properties of osteosarcoma cells by suppression of the AKT/GSK-3/β-catenin pathway. The level of β-catenin expression has been associated with clinical prognosis [97].

Certain natural products will suppress PI3K activity which results in increased GSK-3 activity. This was observed with the neem limonoid nimbolide, obtained from neem tree leaves, in oral cancers. Nimbolide was determined to inhibit cytoprotective autophagy [98,99].

Oridonin is a natural product which has anti-cancer activities. It is a diterpenoid isolated from the leaves of the medicinal herb *Rabdosia rubescens*. Oridonin reduced c-MYC expression which was determined to be mediated by the ubiquitin-proteasome system. F-box and WD repeat domain-containing 7 (Fbw7) protein is an E3 ubiquitin ligase, which regulates c-MYC protein levels. Fbw7 is up-regulated in certain leukemia cells and results in the rapid degradation of c-MYC. In cells with mutations in the WD domain of Fbw7, the oridonin-induced degradation of c-MYC was reduced. Oridonin also induces GSK-3 which is also a priming kinase for Fbw7. This treatment also results in an increase in c-MYC phosphorylated at T58 which targets c-MYC for proteasomal degradation [100]. In this scenario, GSK-3, Fbw7 and oridonin were all acting as tumor suppressors.

Apicidin is a fungal metabolite and it has properties as a histone deacetylase inhibitor. In Apicidin-resistant HA22T hepatocellular carcinoma cells (HCC), the WNT/β-catenin and MMP2 are often activated. This occurs in response to IGF-1R/PI3K/AKT signaling which results in activation of the transcription factor SNAIL but leads to decreased GSK-3 expression. The increased IKKα/β/NF-κB pathway resulted in WNT/β-catenin signaling and cancer growth [101]. Therapies that increase GSK-3 expression may enhance the therapy of apicidin-resistant HCC cells.

Wogonin is a monoflavonoid derived from plants. It inhibits cell growth and induces apoptosis by altering the expression of GSK-3β, the anti-apoptotic X-linked inhibitor of apoptosis protein (XIAP) and induced myeloid leukemia cell differentiation protein (MCL1) molecules and degradation of other molecules such as c-MYC, S-phase kinase-associated protein 2 (SKP2), histone deacetylase 1(HDAC1) and HDAC2 in certain cell types such as the lung cancer A549 cell line [102]. Increased T58-MYC expression, which is now targeted for proteasomal degradation, may be linked to decreased levels of phosphorylated S9-P-GSK-3β (e.g., lowered levels of inactivated GSK-3). Active GSK-3β may phosphorylate c-MYC on residue T58 and contribute to the induction of apoptosis. In this scenario, GSK-3β was acting as a tumor suppressor.

Treatment of lung cancer CSCs with sulforaphane resulted in induction of miR-19 and suppression of GSK-3β and increased WNT/β-catenin expression. Sulforaphane is present in broccoli, cauliflower and other cruciferous vegetables [103].

Butyrate is a short chain fatty acid. It is produced by microbiota in the gut during anaerobic fermentation of fiber. Butyrate can suppress tumor progression by inhibiting histone deacetylase and stimulating apoptosis. miR-22 has roles in regulation of butyrate-induced reactive oxygen species (ROS) release-mediated apoptosis in HCC cells. miR-22 expression was elevated when Huh 7 HCC cells were treated with sodium butyrate. miR-22 and sodium butyrate were determined to increase ROS expression and decrease SIRT-1 expression. Anti-miR-22 was demonstrated to reverse the butyrate-induced effects. Butyrate increased expression of miR-22 which resulted in increased expression of PTEN and active GSK-3 and decreased expression of phosphorylated AKT and β-catenin expression [104]. Thus, in this scenario miR-22 and GSK-3 were with tumor suppressor effects.

Ursolic acid is a pentacyclic triterpenoid isolated from various plants and fruit (e.g., skin of apples) and has anti-cancer activity against multiple cancer types. Ursolic acid inhibits WNT/β-catenin signaling and induces apoptosis in PC-3 prostate cancer cells. Ursolic acid treatment suppressed WNT5/β-catenin expression and enhanced the phosphorylation of GSK-3β. Treatment with the GSK-3β inhibitor SB216763 reversed the induction of apoptosis induced by ursolic acid. Thus, in this scenario, ursolic acid was modifying GSK-3 and WNT5/β-catenin activities and inducing apoptosis. Suppression of GSK-3 activity inhibited the growth inhibitory effects of ursolic acid. Thus, GSK-3 was normally promoting growth in these cells and acting as a tumor promoter [105]. Inactivating GSK-3 with ursolic acid increased apoptosis.

Ursolic acid can also be isolated from *Oldenlandia diffusa*, a plant frequently used in traditional Chinese medicine for treatment of many disorders and maladies including snake bite. Ursolic acid induces cell death in SK-OV-3 ovarian cancer cells by phosphorylation of GSK-3 and apoptosis by activation of caspases 3 and 9, and cleavage of poly (ADP ribose) polymerase (PARP), and suppression of the expression of certain anti-apoptotic genes such as BCL2 and others. Interestingly, ursolic acid treatment suppressed β-catenin degradation and enhanced phosphorylation of GSK-3. Suppression of GSK-3 activity by treatment with the GSK-3 inhibitor SB216763 prevented the cleavage of caspase-3 and PARP normally induced by ursolic acid. Thus, in this scenario, GSK-3 was serving as a tumor promoter and suppression of its activity prevented cancer growth. Ursolic acid normally induces the phosphorylation of GSK-3. Treatment of SK-OV-3 ovarian cancer cells with GSK-3 inhibitors blocked the effects of ursolic acid on the phosphorylation of GSK-3 and induction of apoptosis [106]. Inactivating GSK-3 with ursolic acid increased apoptosis in ovarian cancer cells.

Gambogenic acid is a polyprenylated xanthone isolated from the gamboge family and is used as a nutraceutical. Gambogenic acid reduced phosphorylated AKT and GSK-3 in U251 GBM cells. Gambogenic acid stimulated GSK-3 activity and inhibited growth of glioblastoma cells [107].

## 4. GSK-3 Inhibitors in Cancer Clinical Trials

Table 2 presents a summary of GSK-3 inhibitors that are or have been evaluated in clinical trials/studies.

NCT03678883 is an ongoing clinical trial with 9-ING-41 which will enroll patients with various advanced cancers. The patients will be treated with various chemotherapeutic drugs and 9-ING-41. The title of the trial is: “9-ING-41 in Patients With Advanced Cancers”.

NCT04239092 is a clinical trial with 9-ING-41 not yet started. It is entitled: “Phase 1 Study of 9-ING-41, a Glycogen Synthase Kinase 3 Beta (GSK 3β) Inhibitor, as a Single Agent or With Irinotecan in Pediatric Patients With Refractory Malignancies.” 

NCT01632306 was a clinical trial with LY2090314 and chemotherapy with metastatic pancreatic cancer patients. It was entitled “A Study of LY2090314 and Chemotherapy in Participants With Metastatic Pancreatic Cancer”. It was terminated due to slow enrollment. Unfortunately, no patients were enrolled in the arm with LY2090314 + Gemcitabine + Nab-paclitaxel treatment.

Perhaps as we learn more about the basic biochemical mechanisms of GSK-3 in various cancer types, clinicians and drug companies will start more trials with certain cancer patients and various GSK-3 inhibitors and other drugs.

## 5. Conclusions

GSK-3 is a multifaceted enzyme which phosphorylates numerous protein substrates and often alters the stability of the effected protein. Often, but not always, when a protein is phosphorylated by GSK-3 it is targeted for degradation. Since GSK-3 can phosphorylate so many proteins, these phosphorylation events may result in the suppression of a process (e.g., inhibition on invasion by phosphorylation of the SNAIL transcription factor which normally promotes invasion) or activation of a process (e.g., phosphorylation of p27^Kip-1^ which normally suppresses cell growth and cell growth now occurs after GSK-3 phosphorylation of p27^Kip-1^). This dichotomy of results can in part explain why it is not easy to predict if treatment with GSK-3 inhibitors may or may not be effective in suppressing cancer growth. Numerous GSK-3 inhibitors were designed. GSK-3 is a potential target in cancer due to its roles in various signaling pathways including the NF-κB protein which is often deregulated in cancer and can induce many pro-inflammatory genes which contribute to cancer progression. GSK-3 also plays prominent roles in the EGFR/PI3K/AKT/GSK-3/mTORC1, NF-κB and the WNT/β-catenin pathways. All three of these pathways are often aberrantly regulated in human cancer and tumor progression.

Numerous studies indicated that GSK-3 inhibitors can suppress the growth of certain cancers. However, the potential of GSK-3 inhibitors to be used in treatment of patients with cancer may be more effective by combining GSK-3 inhibitors with either other small molecule inhibitors or chemotherapy or radiotherapy. Many natural products also affect GSK-3 activity. These natural products may represent an approach to treat various conditions as they are often present in our diet or can be added as supplements (nutraceuticals). Thus, the natural products may represent an alternative approach to cancer treatment as they may be less toxic than certain chemotherapeutic drugs.

## Figures and Tables

**Table 1:** GSK-3α and GSK-3β Expression in Different Human Tissue Types.

Tissue Type	GSK-3α	GSK-3β	Ratio GSK-3α/ GSK-3β
Adrenal	12.5 ± 0.7	7.3 ± 0.1	1.7
Appendix	11 ± 1.2	6.2 ± 0.8	1.8
Bone Marrow	10.4 ± 1.1	3.7 ± 0.5	2.8
Brain	26.3 ± 6.8	13.9 ± 1.2	1.9
Colon	10.8 ± 1.3	6.4 ± 0.5	1.7
Duodenum	16.2 ± 0.07	5 ± 0.3	3.2
Endometrium	11.7 ± 0.6	6.5 ± 1.5	1.8
Esophagus	11.9 ± 0.5	5.9 ± 0.6	2
Fat	10 ± 2	5.7 ± 0.6	1.8
Gall bladder	10.8 ± 0.2	6.6 ± 1.3	1.6
Heart	9.6 ± 0.8	6.7 ± 0.7	1.4
Kidney	12.8 ± 1.2	5.9 ± 0.5	2.2
Liver	4.6 ± 0.6	2.8 ± 0.1	1.6
Lung	11.6 ± 1.5	7.9 ± 1.1	1.5
Lymph node	10.5 ± 1.6	5.5 ± 1.4	1.9
Ovary	12.1 ± 0.3	4.6 ± 0.07	2.6
Pancreas	3.1 ± 0.04	1.4 ± 0.05	2.2
Placenta	12.4 ± 1.3	7.7 ± 1.7	1.6
Prostate	12.4 ± 0.6	5.5 ± 0.5	2.3
Salivary gland	5.1 ± 1.1	2.9 ± 0.5	1.8
Skin	11.8 ± 1	7.4 ± 2.3	1.6
Small intestine	14.4 ± 0.7	5.7 ± 0.5	2.5
Spleen	11.6 ± 0.6	7.1 ± 0.9	1.6
Stomach	8.7 ± 2.1	4.9 ± 1.2	1.8
Thyroid	13.3 ± 1.2	9.9 ± 1.7	1.3
Urinary bladder	10.9 ± 0.7	6.5 ± 1.2	1.7

(Reads per Kilobase Million).

**Table 2:** Small Molecule Inhibitors Used to Inhibit GSK-3 Activity in Clinical Trials/Studies.

Molecule	Result	Clinical Trial	Number/Reference
9-ING-41, GSK-3β inhibitor	Ongoing, recruiting	9-ING-41 in Patients With Advanced Cancers, 29 advanced cancer types also including chemotherapeutic drugs.	NCT03678883
9-ING-41, GSK-3β inhibitor	Not yet recruiting	9-ING-41 in Pediatric Patients With Refractory Malignancies, 10 different types of pediatric cancers also including chemotherapeutic drugs	NCT04239092
LY2090314, GSK-3β inhibitor	Terminated, due to slow recruitment	A Study of LY2090314 and Chemotherapy in Participants With Metastatic Pancreatic Cancer	NCT01632306, results are presented on ClinicalTrials.gov
AKT inhibitor AZD5363, which will result in GSK-3 activation, and combination of the PARP inhibitor olaparib	Phase I trial of effects of combining Olaparib and AZD5362. Completed	Trial of Olaparib in Combination With AZD5363 (ComPAKT) (ComPAKT) in advanced cancer patients.	NCT02338622, no results posted.
Combining the EGFR/HER2 inhibitor with the proteasomal inhibitor bortezomib. Lapatinib should inhibit AKT activity which will lead to GSK-3 activity.	Phase I study was terminated due to withdrawal of sponsor support.	A Phase I Study of the HER1, HER2 Dual Kinase Inhibitor, Lapatinib Plus the Proteasomal Inhibitor Bortezomib in Patients With Advanced Malignancies	NCT01497626, [108].
EGFR inhibitor panitumumab in combination with lycopene which is a potent antioxidant. EGFR inhibitor should suppress downstream AKT which lead to GSK-3 activity.	Recruiting	Panitumumab Skin Toxicity Prevention Trial (PaSTo). Colorectal cancer patients.	NCT03167268, no results posted
The protein kinase C beta inhibitor Enzastaurin result in inhibition of AKT which leads to activation of GSK-3. The effects of Enzataurin and the vascular endothelial growth factor A (VEGFa) inhibitor Bevacizumab were examined in advanced or metastatic cancer patients.	Finished Phase I study. 67 patients were evaluable for safety and efficacy. Good results with patients with ovarian cancers. 50.4 % of ovarian cancer patients remained without disease progression after 6 months.	Enzastaurin and Bevacizumab in Treating Patients With Locally Advanced or Metastatic Cancer	NCT00550927, [58].
To determine effects of combination of enzataurin and bevacizumab in adults with glioma.	Finished Phase II study with 81 patients with glioblastomas (GBM, n = 40) and anaplastic gliomas (AG, n = 41). Early response was associated with longer progression free survival for glioblastomas. Combined treatment was well tolerated, and survival time was similar to that observed in patients treated with bevacizumab.	Phase II study with enzastaurin (LY317615) in combination with bevacizumab in adults with recurrent malignant gliomas.	NCT00586508, [59].
Effects of combining Enzastaurin (LY317615) With Carboplatin on recurrent glioma patients	Completed	A Phase I Trial of Enzastaurin (LY317615) in Combination With Carboplatin in Adults With Recurrent Gliomas	NCT01445119, No results with combining Enzastaurin (LY317615) with Carboplatin.
Effects of Trametinib MEK inhibitor and pan AKT inhibitor (GSK2141795) treatment in melanoma. Suppression of AKT should result in increased levels of active GSK-3	Completed, Phase II clinical study did not reveal any clinical benefit of trametinib and GSK2141795 treatment in melanoma patients with *NRAS* mutations or wild-type melanoma. GSK 2141795 inhibited phosphorylation of GSK-3β.	Trametinib With GSK2141795 in BRAF Wild-type Melanoma	NCT01941927, [109].
Treatment of humans and mouse model of recurrent GBM with temozolomide (TMZ) and other drugs which suppress GSK-3β (cimetidine, lithium, olanzapine, and valproate, (CLOVA) cocktail. The safety and efficacy of the CLOVA cocktail) in combination with TMZ were performed to human and murine studies.	Inhibition of active GSK-3β in the tumor resulted in increased patient survival. The combination of TMZ and the CLOVA cocktail significantly inhibited cell invasion and TMZ increased survival compared to patients treated with TMZ alone. Active GSK-3β was associated with a poor prognosis	Clinical study in Japan completed with 7 GBM patients.	[110].

## Supplementary Materials

Supplementary Materials can be found at the following link: https://www.mdpi.com/2073-4409/9/5/1110/s1: Table S1: Examples of Involvement of GSK-3 Isoforms in Cancer; Table S2: Examples of Preclinical Studies with GSK-3 Inhibitors and Nutraceuticals/Natural Products Involving Cancer Models.
