# Supplementary material for: Targeting GSK3 and Associated Signaling Pathways Involved in Cancer
Source: Cells. 2020 Apr 30;9(5):1110. doi: 10.3390/cells9051110 (PMC7290852; doi:10.3390/cells9051110)
Supplement: Supplementary file 1 [file cells-09-01110-s001.pdf]

Table S1. Examples of Involvement of GSK-3 Isoforms in Cancer.

Cancer Type (alphabetical)	GSK-3 Isoform	Function	Type of Study	Reference
Bladder cancer	GSK-3β	Prognostic marker and therapeutic target. Inhibition of GSK-3 resulted in apoptosis. GSK-3 was serving as a tumor promoter. Aberrant nuclear accumulation of GSK-3β in 62% and 91% of noninvasive and invasive human urothelial carcinomas. GSK-3β nuclear staining was associated with poor prognosis.	Human tumor samples and in vitro	[1]
Brain cancer	GSK-3β	Brain-derived neutrophilic factor/TrkB induced phosphorylation of GSK-3β which resulted in its inactivation and contributed to chemotherapeutic drug resistance. GSK-3β was acting as a tumor suppressor.	In vitro	[2]
Brain cancer	GSK-3β	Inhibition of AKT mediated phosphorylation of GSK-3β by an AKT inhibitor reduced cell growth. GSK-3β was acting as a tumor suppressor.	In vitro	[3]
Brain cancer	GSK-3β	GSK3β was linked with increased expression of TP53 and $p21^{Cip-1}$ in glioblastoma cells with wild-type p53 and with decreased Rb phosphorylation and expression of cyclin-dependent kinase 6, Treatment with GSK-3 inhibitor AR-A014418 sensitized GMB cells to temozolomide. GSK-3β was functioning as a tumor promoter.	Human tumor samples, in vitro studies.	[4]
Brain cancer	GSK-3β	Expression of high levels of GSK-3β was associated with poor prognosis. Treatment with a combination of temozolomide other drugs used to treat brain cancer improved prognosis. GSK-3β was acting as a tumor promoter.	In vitro, in vivo, clinical trial, 7 patients in clinical study	[5]
Brain cancer		Suppression of GSK-3β by miR-101 restored sensitivity to temozomide in brain cancer.	In vitro, in vivo	[6]

		GSK-3β was acting as a tumor promoter.		
Breast cancer	GSK-3β	GSK-3β expression was associated with MCL1 expression and inactivation. GSK-3β was acting as a tumor suppressor. High MCL1 expression was associated with poor prognosis and high P-GSK-3β (inactive) expression.	Human tumor samples (125 breast cancer), in vitro	[7]
Breast cancer	GSK-3β	High GSK-3β expression was associated with reduced distant relapse-free survival (DRFS). Tissue microarrays of 1,686 patients, low expression in 36%, high expression in 38%. GSK-3β was acting as a tumor promoter.	Human tumor samples.	[8]
Breast cancer	GSK-3β	Inhibition of GSK-3β inhibited tumor growth GSK-3β was acting as a tumor promoter.	In vitro, in vivo	[9]
Breast cancer	GSK-3β	miR-34a binding to the *PRKD1* suppressed cancer stemness through the GSK3/β-catenin signaling pathway. GSK-3 was acting as a stemness suppressor.	In vitro, in vivo	[10]
Breast cancer	GSK-3β	GSK-3 inhibition by the human THUMP domain-containing protein 1 (THUMPD1)/AKT resulted in SNAIL activation. GSK-3 was acting as a tumor suppressor.	In vitro, in vivo	[11]
Cervical cancer	GSK-3β	High expression of forkhead box M1 (FOXM1) transcription factor was associated with poor prognosis and it activated AKT and inactivated GSK-3β which resulted in higher SNAIL activity and poor prognosis. GSK-3β was acting as a tumor suppressor.	In vitro, human tumor samples	[12]
Colorectal cancer	GSK-3β	Nuclear accumulation of GSK-3β was observed in 39% (33/85) and associated with short overall survival, larger tumor size, distant metastasis and loss of membranous β-catenin. This loss was present in 37% and associated with poor survival. Nuclear expression of GSK-3β and loss of membrane β-catenin were present in CRC with worse	Human tissue microarrays	[13]

		prognosis. GSK-3β was functioning as a tumor promoter,		
Colorectal cancer	GSK-3β	GSK-3β increased NF-κB expression, inhibition of GSK-3 inhibited growth. GSK-3β was serving as a tumor promoter.	Human tumors and *in vitro* studies	[14]
Colorectal cancer	GSK-3β	CXCL5 induced ERK/ELK1/SNAIL and AKT/β-catenin, inhibited GSK-3β and promoted cancer metastasis. GSK-3β was acting as a tumor promoter.	In vitro, in vivo. chemokine ELISA arrays from CRC patients	[15]
Gastric cancer	GSK-3β	P-GSK-3β (T216, active) was expressed in 46% of cases and associated with a good prognosis. GSK-3β was acting as a tumor suppressor.	Human tissue arrays containing 281 gastric cancer specimens and in vitro studies	[16]
Gastric cancer	GSK-3β	Higher GSK-3β levels were associated with a better prognosis. GSK-3β was acting as a tumor suppressor.	Gene expression profiling in 63 tumors	[17]
Hepatocellular carcinoma	GSK-3β	S9-P-GSK-3β was over-expressed in 50% of tumor tissues and was associated with a poor prognosis. GSK-3β was acting as a tumor suppressor.	178 patients with HCC after curative partial hepatectomy	[18]
Hepatocellular carcinoma	GSK-3β	Protein arginine methyltransferase 9 (PRMT9) activation of PI3K/AKT resulted in decreased GSK-3β activity and increased SNAIL signaling. GSK-3β was acting as a tumor suppressor	In vitro, in vivo, human tumor samples	[19]
Laryngeal Cancer	GSK-3β	Suppression of miR-27a interaction with GSK-3β altered laryngeal differentiation in response to retinoic acid treatment. GSK-3β. GSK-3β was acting as a tumor suppressor.	In vitro, human tumor samples	[20]
Laryngeal Cancer	GSK-3β	Alterations in the Tat-interacting protein 30 (TIP30) tumor suppressor expression resulted in activation of AKT, inactivation of GSK-3β, deregulation of β-catenin and poor prognosis. Low TIP30 staining was observed in 43.8% of patient samples while minimal TIP30 staining in non-tumor cells was observed in	In vitro, human tumor samples, 105 laryngeal carcinomas	[21]

		25.7% of samples. GSK-3β was acting as a tumor suppressor.		
Leukemia	GSK-3β and GSK-3α	Genetic deletion of GSK-3β in mice led to myelodysplastic disease syndrome (MDS), subsequent deletion of GSK-3α led to AML. Different roles of GSK-3α and GSK-3β in MDS progression into AML. GSK-3α and GSK-3β were acting as tumor suppressors.	Gene knock out studies in mice, gene profiling.	[22]
Leukemia	GSK-3α and GSK-3β	GSK-3 stimulated acute lymphoblastic leukemia with mixed-lineage leukemia gene (MLL) growth by destabilization of the cyclin-dependent kinase inhibitor p27(Kip1). GSK-3 promoted growth, GSK-3 was acting as a tumor promoter.	In vitro, in vivo, in human AML patients	[23]
Leukemia	GSK-3α	GSK-3α was a target in AML. GSK-3α was serving as a tumor promoter.	Chemical small molecule screening, in vitro, in vivo	[24]
Leukemia (AML)	GSK-3α and GSK-3β	GSK-3α and GSK-3β phosphorylation leading to their inhibition correlated with poor prognosis. S21-P-GSK3α and S9-P-GSK-3β positively correlated with phosphorylation of AKT, BAD, and P70S6K, and negatively correlated with β-catenin and FOXO3A. GSK-3α and GSK-3β were serving as tumor suppressors	In vitro, human patient samples, reverse phase protein analysis (RPPA) in a cohort of 511 AML patients	[25]
Leukemia (Natural Killer Cells cytotoxic to AML)	GSK-3β and GSK-3α	(GSK-3β) expression was elevated in AML-NK cells and decreased their activity as NK cells. Inhibition of GSK-3 restored NK cytotoxicity by increasing TNF-α production. GSK-3 was serving as a tumor suppressor.	In vitro, in vivo	[26]
Lung cancer	GSK-3β	High levels of TGFβ induced integrin β3/AKT, inhibited GSK-3β activity, and induced SNAIL activity and promoted metastatic potential. GSK-3β was acting as a tumor suppressor.	In vitro, in vivo, clinical data base	[27]

Lung cancer	GSK-3α	CREB induced GSK-3α which promoted lung cancer cell growth. GSK-3α was acting as a tumor promoter.	In vitro, in vivo, human tumors	[28]
Lung cancer	GSK-3α and GSK-3β	Tivantinib was initially thought to be a c-MET inhibitor. Subsequently, GSK-3α and GSK-3β were determined to be targets of tivantinib in lung cancer cells. GSK-3α and GSK-3β were acting as tumor promoter	In vitro	[29]
Lung cancer (non-small cell)	GSK-3α and GSK-3β	GSK-3β levels were elevated in 41% of human NSCLC samples and led to increased proliferation in comparison to normal tissues. GSK-3β was acting as a tumor promoter.	In vitro, in vivo, 29 human tumor specimens	[30]
Melanoma	GSK-3α	Elevated expression of GSK-3α in 72% of samples, but not GSK-3β. 80% of tumors expressed elevated levels of catalytically active phosphorylated GSK-3α (Y279-P-GSK-3α), but not phosphorylated GSK3β (Y216-P-GSK-3β). Inhibition of GSK-3α induced apoptotic death to retard tumorigenesis. GSK-3α was acting as a tumor promoter.	In vitro, in vivo, 39 human tumor samples.	[31]
Melanoma	GSK-3β	Neuron navigator 2 (NAV2) inhibited GSK-3β which increased β-catenin and SNAIL activity. GSK-3β was acting as a tumor suppressor.	In vitro, in vivo, human tumor samples	[32]
Myeloma	GSK-3α and GSK-3β	Treatment with Thiadiazolidinone (TDZD; a GSK-3 non-competitive inhibitor) resulted in Forkhead transcription factors (FOXO3a) activation. TDZD induced apoptosis in primary myeloma cells but not in normal CD34 cells. GSK-3 was acting as a tumor promoter.	In vitro, human myeloma cells, primary hematopoietic cells	[33]
Neuroblastoma	GSK-3β	Inhibition of GSK-3β with 9-ING-41 suppressed growth via inhibition of XIAP. GSK-3β was acting as a tumor promoter.	In vitro, in vivo	[34]
Oral Cancer	GSK-3β	AKT and GSK-3β expression was associated with a poor prognosis. Phosphorylated	Human tumor specimens (118 patient samples	[35]

		GSK-3β (inactive) was associated with cervical lymph node (CLN) metastasis. GSK-3β was acting as a tumor suppressor.	and normal controls).	
Oral squamous cell cancer	GSK-3α and GSK-3β	Links between GSK-3α and GSK-3β and cyclin D1 and TP53. Inactive GSK-3β was expressed at higher levels than inactive GSK-3α. Inactive GSK-3β was detected at increased percentages in older patients (40->70 years old) than younger patients (<40 years old). GSK-3β was acting as a tumor suppressor.	In vitro, 179 human patient samples	[36]
Osteosarcoma	GSK-3β	The P2X7 receptor promoted PI3K/AKT and β-catenin activity and inhibited GSK-3β. GSK-3β was acting as a tumor suppressor.	In vitro, in vivo, human tumor samples	[37]
Ovarian cancer	GSK-3β	GSK-3 expression was associated with increased tumor growth, poor prognosis and chemoresistance. GSK-3 was functioning as a tumor promoter.	In vitro, in vivo, 71 human tumor samples.	[38]
Ovarian cancer	GSK-3β	Constitutively active GSK-3β induced entry into the S phase, increased cyclin D1 expression and facilitated the proliferation of ovarian cancer cells. GSK-3 inhibition prevented the tumor formation of the tumor in nude mice. GSK-3 was acting as a tumor promoter.	In vitro, in vivo	[39]
Pancreatic cancer	GSK-3α and GSK-3β	GSK-3 promoted NF-κB activity. GSK-3β may have been the more important isozyme in regulating in NF-κB. GSK-3β was acting as a tumor promoter.	Human tumors and in vitro studies.	[40]
Pancreatic cancer	GSK-3β	Inhibition of GSK-3 activity caused stabilization of β-catenin activity. GSK-3β expression was a strong prognosticator in PDAC. High expression of GSK-3β was associated with better survival. PDAC Patients with GSK-3β expression > than the third quartile (Q3) had a 46% reduced risk of dying of	Immuno-fluorescence on human tumor microarray from 163 patients.	[41]

		pancreatic cancer. GSK-3β was acting as a tumor suppressor.		
Prostate Cancer	Both	GSK-3α and GSK-3β were detected at higher levels in 25/79 and 24/79 tumor samples respectively, in comparison to normal prostatic tissue. GSK-3α was elevated in low Gleason sum score tumors while GSK-3β was expressed in high Gleason tumors, and both isoforms correlated with high expression of the androgen receptor (AR). Treatment with a GSK-3 inhibitor suppressed proliferation. GSK-3 was functioning as a tumor promoter.	In vitro, in vivo and in 79 human tumor samples	[42]
Renal Cell Carcinoma	GSK-3β	miR-199a downregulated GSK-3β and suppressed growth of RCC. GSK-3β was acting as a tumor promoter.	Human tumor samples and in vitro.	[43]
Renal Cell Carcinoma	GSK-3β	miR-203a targeting GSK-3β was detected at high levels in RCC and associated with a poor prognosis. miR-203a was overexpressed in 27 of 40 (68%) RCC patient samples. GSK-3β was acting as a tumor suppressor.	In vitro, 40 RCC tumor samples.	[44]
Thyroid carcinomas	GSK-3α and GSK-3β	Junctional adhesion molecule A (JAM-A) was downregulated in anaplastic thyroid carcinomas and resulted in increased GSK-3α, GSK-3β, and TP53 phosphorylation.	Human tissue arrays	[45]
Tongue (oral) cancer	GSK-3β	GSK-3β was detected at lower levels in 39% of patient samples in comparison to normal epithelial cells and was associated with reduced survival. In contrast, cyclinD, a target of GSK-3β was detected at higher levels in 65.9% of samples and was associated with a poor prognosis. GSK-3β was acting as a tumor suppressor	41 Human tissue samples, immunohisto-chemistry.	[46]

References:

1. Naito, S.; Bilim, V.; Yuuki, K.; Ugolkov, A.; Motoyama, T.; Nagaoka, A.; Kato, T.; Tomita, Y. Glycogen synthase kinase-3β: A prognostic marker and a potential therapeutic target in human bladder cancer. *Clin Cancer Res* **2010**, *16*, 5124–5132.
2. Li, Z.; Tan, F.; Thiele, C.J. Inactivation of glycogen synthase kinase-3β contributes to brain-derived neutrophic factor/TrkB-induced resistance to chemotherapy in neuroblastoma cells. *Mol Cancer Ther* **2007**, *6*, 3113-3121.
3. Atkins, R.J.; Dimou, J.; Paradiso, L.; Morokoff, A.P.; Kaye, A.H.; Drummond, K.J.; Hovens, C.M. Regulation of glycogen synthase kinase-3 beta (GSK-3β) by the Akt pathway in gliomas. *J Clin Neurosci* **2012**, *19*, 1558–1563.
4. Miyashita, K.; Kawakami, K.; Nakada, M.; Mai, W.; Shakoori, A.; Fujisawa, H.; Hayashi, Y.; Hamada, J.; Minamoto, T. Potential therapeutic effect of glycogen synthase kinase 3β inhibition against human glioblastoma. *Clin Cancer Res* **2009**, *15*, 887–897.
5. Furuta, T.; Sabit, H.; Dong, Y.; Miyashita, K.; Kinoshita, M.; Uchiyama, N.; Hayashi, Y.; Hayashi, Y.; Minamoto, T.; Nakada, M. Biological basis and clinical study of glycogen synthase kinase- 3β-targeted therapy by drug repositioning for glioblastoma. *Oncotarget* **2017**, *8*, 22811–22824.
6. Tian, T.; Mingyi, M.; Qiu, X.; Qiu, Y. MicroRNA-101 reverses temozolomide resistance by inhibition of GSK3β in glioblastoma. *Oncotarget* **2016**, *7*, 79584–79595.
7. Ding, Q.; He, X.; Xia, W.; Hsu, J.M.; Chen, C.; Li, L.Y.; Lee, D.F.; Yang, J.Y.; Xie, X.; Liu, J.C.; Hung, M.C. Myeloid cell leukemia-1 inversely correlates with glycogen synthase kinase-3β activity and associates with poor prognosis in human breast cancer. *Cancer Res* **2007**, *67*, 4564–4571.
8. Quintayo, M.A.; Munro, A.F.; Thomas, J.; Kunkler, I.H.; Jack, W.; Kerr, G.R.; Dixon, J.M.; Chetty, U.; Bartlett, J.M.S. GSK3β and cyclin D1 expression predicts outcome in early breast cancer patients. *Breast Cancer Res Treat* **2012**, *136*,161-168.
9. Ugolkov, A.; Gaisina, I.; Zhang, J.S.; Billadeau, D.D.; White, K.; Kozikowski, A.; Jain, S.; Cristofanilli, M.; Giles, F.; O'Halloran, T.; Cryns, V.L.; Mazar, A.P. GSK-3 inhibition overcomes chemoresistance in human breast cancer. *Cancer Lett* **2016**, *380*, 384–392.
10. Kim, D.Y.; Park, E.Y.; Chang, E.S.; Kang, H.G.; Koo, Y.; Lee, E.J.; Ko, J.Y.; Kong, H.K.; Chun, K.H.; Park, J.H. A novel miR-34a target, protein kinase D1, stimulates cancer stemness and drug resistance through GSK3/β-catenin signaling in breast cancer. *Oncotarget* **2016**, *7*, 14791–14802.
11. Zhang, X.; Jiang, G.; Sun, M.; Zhou, H.; Miao, Y.; Liang, M.; Wang, E.; Zhang, Y. Cytosolic THUMPD1 promotes breast cancer cells invasion and metastasis via the AKT-GSK3-Snail pathway. *Oncotarget* **2017**, *8*, 13357–13366.
12. He, S.Y.; Shen, H.W.; Xu, L.; Zhao, X.H.; Yuan, L.; Niu, G.; You, Z.S.; Yao, S.Z. FOXM1 promotes tumor cell invasion and correlates with poor prognosis in early-stage cervical cancer. *Gynecol Oncol* **2012**, *127*, 601-610.
13. Salim, T.; Sjölander, A.; Sand-Dejmek, J. Nuclear expression of Glycogen synthase kinase-3β and lack of membranous β-catenin is correlated with poor survival in colon cancer. *Int J Cancer* **2013**, *133*, 807–815.
14. Shakoori, A.; Ougolkov, A.; Zhi, W.Y.; Zhang, B.; Modarressi, M.H.; Billadeau, D.D.; Mai, M.; Takahashi, Y.; Minamoto, T. Deregulated GSK3β activity in colorectal cancer: Its association with tumor cell survival and proliferation. *Biochem Biophys Res Commun* **2005**, *334*, 1365–1373.
15. Zhao, J.; Ou, B.; Han, D.; Wang, P.; Zong, Y.; Zhu, C.; Liu, D.; Zheng, M.; Sun, J.; Feng, H.; Lu, A. Tumor-derived CXCL5 promotes human colorectal cancer metastasis through activation of the ERK/Elk-1/Snail and AKT/GSK3β/β-catenin pathways. *Mol Cancer* **2017**, *16*, 70.
16. Cho, Y.J.; Kim, J.H.; Yoon, J.; Cho, S.J.; Ko, Y.S.; Park, J.W.; Lee, H.S.; Lee, H.E.; Kim, W.H.; Lee, B.L. Constitutive activation of glycogen synthase kinase-3β correlates with better prognosis and cyclin-dependent kinase inhibitors in human gastric cancer. *BMC Gastroenterol* **2010**, *10*, 91.
17. Bauer, L.; Langer, R.; Becker, K.; Hapfelmeier, A.; Ott, K.; Novotny, A.; Höfler, H.; Keller, G. Expression profiling of stem cell-related genes in neoadjuvant-treated gastric cancer: A NOTCH2, GSK3B and β-catenin gene signature predicts survival. *PLoS One* **2012**, *7*, e44566.
18. Qiao, G.; Le, Y.; Li, J.; Wang, L.; Shen, F. Glycogen synthase kinase-3β is associated with the prognosis of hepatocellular carcinoma and may mediate the influence of type 2 diabetes mellitus on hepatocellular carcinoma. *PLoS One* **2014**, *9*, e105624.
19. Jiang, H.; Zhou, Z.; Jin, S.; Xu, K.; Zhang, H.; Xu, J.; Sun, Q.; Wang, J.; Xu, J. PRMT9 promotes hepatocellular carcinoma invasion and metastasis via activating PI3K/Akt/GSK-3β/Snail signaling. *Cancer Sci* **2018**, *109*, 1414–1427.
20. Chen, S.; Sun, Y.Y.; Zhang, Z.X.; Li, Y.H.; Xu, Z.M.; Fu, W.N. Transcriptional suppression of microRNA-27a contributes to laryngeal cancer differentiation via GSK-3β-involved Wnt/β-catenin pathway. *Oncotarget* **2017**, *8*, 14708-14718.

21. Zhu, M.; Yin, F.; Yang, L.; Chen, S.; Chen, R.; Zhou, W.; Fan, X.; Jia, R.; Zheng, H.; Zhao, J.; Guo, Y. Contribution of TIP30 to chemoresistance in laryngeal carcinoma. *Cell Death Dis* **2014**, *5*, e1468.
22. Guezguez, B.; Almakadi, M.; Benoit, Y.D.; Shapovalova, Z.; Rahmig, S.; Fiebig-Comyn, A.; Casado, F.L.; Tanasijevic, B.; Bresolin, S.; Masetti, R.; Doble, B.W.; Bhatia, M. GSK3 Deficiencies in Hematopoietic Stem Cells Initiate Pre-neoplastic State that Is Predictive of Clinical Outcomes of Human Acute Leukemia. *Cancer Cell* **2016**, *29*, 61–74.
23. Wang, Z.; Smith, K.S.; Murphy, M.; Piloto, O.; Somervaille, T.C.P.; Cleary, M.L. Glycogen synthase kinase 3 in MLL leukaemia maintenance and targeted therapy. *Nature* **2008**, *455*, 1205–1209.
24. Banerji, V.; Frumm, S.M.; Ross, K.N.; Li, L.S.; Schinzel, A.C.; Hahn, C.K.; Kakoza, R.M.; Chow, K.T.; Ross, L.; Alexe, G.; Tolliday, N.; Inguilizian, H.; Galinsky, I.; Stone, R.M.; DeAngelo, D.J.; Roti, G.; Aster, J.C.; Hahn, W.C.; Kung, A.L.; Stegmaier, K. The intersection of genetic and chemical genomic screens identifies GSK-3α as a target in human acute myeloid leukemia. *J Clin Invest* **2012**, *122*, 935–947.
25. Ruvolo, P.P.; Qiu, Y.H.; Coombes, K.R.; Zhang, N.; Neeley, E.S.; Ruvolo, V.R.; Hail, N.; Borthakur, G.; Konopleva, M.; Andreeff, M.; Kornblau, S.M. Phosphorylation of GSK3α/β correlates with activation of AKT and is prognostic for poor overall survival in acute myeloid leukemia patients. *BBA Clin* **2015**, *4*, 59–68.
26. Parameswaran, R.; Ramakrishnan, P.; Moreton, S.A.; Xia, Z.; Hou, Y.; Lee, D.A.; Gupta, K.; Delima, M.; Beck, R.C.; Wald, D.N. Repression of GSK3 restores NK cell cytotoxicity in AML patients. *Nat Commun* **2016**, *7*, 11154.
27. Bae, G.Y.; Hong, S.K.; Park, J.R.; Kwon, O.S.; Kim, K.T.; Koo, J.H.; Oh, E.; Cha, H.J. Chronic TGFβ stimulation promotes the metastatic potential of lung cancer cells by Snail protein stabilization through integrin β3-Akt-GSK3β signaling. *Oncotarget* **2016**, *7,* 25366-25376.
28. Park, S.A.; Lee, J.W.; Herbst, R.S.; Koo, J.S. GSK-3α is a novel target of CREB and CREB-GSK-3α signaling participates in cell viability in lung cancer. *PLoS One* **2016**, *11*, e0153075.
29. Remsing Rix, L.L.; Kuenzi, B.M.; Luo, Y.; Remily-Wood, E.; Kinose, F.; Wright, G.; Li, J.; Koomen, J.M.; Haura, E.B.; Lawrence, H.R.; Rix, U. GSK3 alpha and beta are new functionally relevant targets of tivantinib in lung cancer cells. *ACS Chem Biol* **2014**, *9*, 353–358.
30. Vincent, E.E.; Elder, D.J.E.; O'Flaherty, L.; Pardo, O.E.; Dzien, P.; Phillips, L.; Morgan, C.; Pawade, J.; May, M.T.; Sohail, M.; Hetzel, M.R.; Seckl, M.J.; Tavaré, J.M. Glycogen synthase kinase 3 protein kinase activity is frequently elevated in human non-small cell lung carcinoma and supports tumour cell proliferation. *PLoS One* **2014**, *9*, e114725.
31. Madhunapantula, S.V.; Sharma, A.; Gowda, R.; Robertson, G.P. Identification of glycogen synthase kinase 3α as a therapeutic target in melanoma. *Pigment Cell Melanoma Res* **2013**, *26*, 886–99.
32. Hu, W.; Li, X.; Cheng, R.; Ke, J.; Liu, Y.; Ma, M.; Cao, Y.; Liu, D. NAV2 facilitates invasion of cutaneous melanoma cells by targeting SNAI2 through the GSK-3β/β-catenin pathway. *Arch Dermatol Res* **2019**, *311*, 399–410.
33. Zhou, Y.; Uddin, S.; Zimmerman, T.; Kang, J.A.; Ulaszek, J.; Wickrema, A. Growth control of multiple myeloma cells through inhibition of glycogen synthase kinase-3. *Leukemia and Lymphoma* **2008**, *49*, 1945–53.
34. Ugolkov, A.V.; Bondarenko, G.I.; Dubrovskyi, O.; Berbegall, A.P.; Navarro, S.; Noguera, R.; O'Halloran, T.V.; Hendrix, M.J.; Giles, F.J.; Mazar, A.P. 9-ING-41, a small-molecule glycogen synthase kinase-3 inhibitor, is active in neuroblastoma. *Anticancer Drugs* **2018**, *29*, 717–724.
35. Matsuo, F.S.; Andrade, M.F.; Loyola, A.M.; da Silva, S.J.; Silva, M.J.B.; Cardoso, S.V.; de Faria, P.R. Pathologic significance of AKT, mTOR, and GSK3β proteins in oral squamous cell carcinoma-affected patients. *Virchows Arch* **2018**, *472*, 983–997.
36. Mishra, R.; Nagini, S.; Rana, A. Expression and inactivation of glycogen synthase kinase 3 alpha/ beta and their association with the expression of cyclin D1 and p53 in oral squamous cell carcinoma progression. *Mol Cancer* **2015**, *14*, 20.
37. Zhang, Y.; Cheng, H.; Li, W.; Wu, H.; Yang, Y. Highly-expressed P2X7 receptor promotes growth and metastasis of human HOS/MNNG osteosarcoma cells via PI3K/Akt/GSK3β/β-catenin and mTOR/HIF1α/VEGF signaling. *Int J Cancer* **2019**, *145*, 1068–1082.
38. Fu, Y.; Wang, X.; Cheng, X.; Ye, F.; Xie, X.; Lu, W. Clinicopathological and biological significance of aberrant activation of glycogen synthase kinase-3 in ovarian cancer. *Onco Targets Ther* **2014**, *7*, 1159–1168.
39. Cao, Q.; Lu, X.; Feng, Y.J. Glycogen synthase kinase-3β positively regulates the proliferation of human ovarian cancer cells. *Cell Res* **2006**, *16*, 671–677.
40. Mamaghani, S.; Patel, S.; Hedley, D.W. Glycogen synthase kinase-3 inhibition disrupts nuclear factor-kappaB activity in pancreatic cancer, but fails to sensitize to gemcitabine chemotherapy. *BMC Cancer* **2009**, *9*, 132.

41. Ben-Josef, E.; George, A.; Regine, W.F.; Abrams, R.; Morgan, M.; Thomas, D.; Schaefer, P.L.; DiPetrillo, T.A.; Fromm, M.; Small, W.; Narayan, S.; Winter, K.; Griffith, K.A.; Guha, C.; Williams, T.M. Glycogen Synthase Kinase 3 Beta Predicts Survival in Resected Adenocarcinoma of the Pancreas. *Clin Cancer Res* **2015**, *21*, 5612–5618.
42. Darrington, R.S.; Campa, V.M.; Walker, M.M.; Bengoa-Vergniory, N.; Gorrono-Etxebarria, I.; Uysal-Onganer, P.; Kawano, Y.; Waxman, J.; Kypta, R.M. Distinct expression and activity of GSK-3α and GSK-3β in prostate cancer. *Int J Cancer* **2012**, *131*, E872–83.
43. Tsukigi, M.; Bilim, V.; Yuuki, K.; Ugolkov, A.; Naito, S.; Nagaoka, A.; Kato, T.; Motoyama, T.; Tomita, Y. Re-expression of miR-199a suppresses renal cancer cell proliferation and survival by targeting GSK-3β. *Cancer Lett* **2012**, *315*, 189–197.
44. Hu, G.; Lai, P.; Liu, M.; Xu, L.; Guo, Z.; Liu, H.; Li, W.; Wang, G.; Yao, X.; Zheng, J.; Xu, Y. miR-203a regulates proliferation, migration, and apoptosis by targeting glycogen synthase kinase-3β in human renal cell carcinoma. *Tumor Biol* **2014**, *35*, 11443–11453.
45. Orlandella, F.M.; Mariniello, R.M.; Iervolino, P.L.C.; Auletta, L.; De Stefano, A.E.; Ugolini, C.; Greco, A.; Mirabelli, P.; Pane, K.; Franzese, M.; Denaro, M.; Basolo, F.; Salvatore, G. Junctional adhesion molecule-A is down-regulated in anaplastic thyroid carcinomas and reduces cancer cell aggressiveness by modulating p53 and GSK3 α/β pathways. *Mol Carcinog* **2019**, *58*, 1181–1193.
46. Goto, H.; Kawano, K.; Kobayashi, I.; Sakai, H.; Yanagisawa, S. Expression of cyclin D1 and GSK-3beta and their predictive value of prognosis in squamous cell carcinomas of the tongue. *Oral Oncol* **2002**, *38*, 549–556.

Table S2. Examples of Preclinical Studies with GSK-3 Inhibitors and Nutraceuticals/Natural Products Involving Cancer Models

Molecule	Result	Reference
**GSK-3 Inhibitors**		
Lithium chloride	Lithium chloride inhibited GSK-3 which suppressed proliferation in Eca-109 human esophageal cancer cells. GSK-3 was functioning as a tumor promoter.	[1]
AR-A014418	Treatment with GSK-3β inhibitor AR-A014418 sensitized GMB cells to temozolomide. GSK-3β was functioning as a tumor promoter.	[2]
BIO	BIO induced apoptosis, cell cycle arrest in glioblastoma cells.	[3]
Tideglusib, AZD1080, and BIO	These GSK-3 inhibitors suppressed GSK-3 mediated phosphorylation of substrates involved in proliferation such as c-MYC in KRAS-dependent tumors.	[4]
ABC1183	ABC1183 inhibited GSK-3α and GSK-3β. ABC1183 inhibited the growth of a numerous cancer cell lines by decreasing cell survival by inducing G_2/M arrest by altering GSK-3 and WNT/β-catenin signaling.	[5]
SB21673	SB21673 inhibits GSK-3α and GSK-3β. c-JUN degradation was enhanced by SB21673 and breast cancer tumorigenesis was inhibited.	[6]
SB216763, GSK inhibitor XIII, and AR-A014418	SB216763 and the GSK inhibitor III suppressed AR-transcriptional activity as well as AR expression in prostate cancer cells. In contrast, AR-A014418 stimulated proliferation.	[7]
Lithium chloride, SB216763, and GSK-3 IX (BIO)	Treatment of MLL LSC with GSK-3 inhibitors resulted in reversion of MLL LSCs to a pre-LSC stage and reduced their growth.	[8]
GSK-3 IX (BIO) and SB216763	Inhibition of GSK-3 suppressed maintenance of MLL leukemia.	[9]
GSK3-IX	The GSK-3α and GSK-3β inhibitor GSK3-IX inhibited MLL leukemia maintenance and growth.	[9]
GS87	GS87 is a novel GSK-3 inhibitor that was isolated upon screening for more optimal effective inhibitors that induce AML differentiation. GS87 inhibits both GSK-3α and GSK-3β.	[10]
Thiadiazolidinone (TDZD)	TDZD is a non-competitive inhibitor of GSK-3. Treatment of human myeloma cells with TDZD resulted in apoptosis in primary myeloma cells but not in normal CD34 cells.	[11]
**Combination of GSK-3 inhibitors with immunotherapy**		
SB415286 and CD8+ CTLs	GSK-3 inhibitor treatment of CD8+ T cells inhibited TBX21 (T-bet) expression and decreased PD-1 expression and increased cytolytic T cell responses.	[12]
LY2090314, tideglusib, SB415286 GSK-3 inhibitors and NK cells	Treatment of NK cells with GSK-3 inhibitors LY2090314, tideglusib or SB415286, increased TNF-α levels and cytotoxicity towards AML cells.	[13]
SB216763 and GMB-specific CAR-T cells	Treatment with GSK-3 inhibitor of antigen specific CAR-T cells lowered PD-1 expression and promoted long term survival, memory and tumor elimination.	[14]
Enzastaurin	Enzastaurin was initially developed as a PKC-β inhibitor. One of its targets is GSK-3. It has been examined in clinical	[15]

	studies with various cancer types, often in combination with bevacizumab.	
SB415286 or LiCl and TRAIL	Inhibition of GSK-3 enhanced the induction of apoptosis mediated by TRAIL in gastric cancer cells.	[16]
**Combination of GSK-3 inhibitors with chemotherapy**		
CHIR99021 and paclitaxel	Effects of combination of the GSK-3 inhibitor CHIR99021 and paclitaxel on lung cancer.	[17]
SB415286, RO 318220, lithium chloride and paclitaxel	SB415286 inhibits both GSK-3α and GSK-3β. RO 318220 inhibits PKC and GSK-3. More mitotic arrest was observed when GSK-3 inhibitors were combined with paclitaxel than in the absence of the GSK-3 inhibitors.	[18]
LY2090314 and nab-paclitaxel	LY2090314 suppressed TAK1 levels. LY2090314 plus nab-paclitaxel combined treatment increased the survival of mice in orthotopic pancreatic tumor models.	[19]
AR-A01441, TDZD-8, 9-ING-41 and Camptosar	AR-A01441, TDZD-8, and 9-ING-41 suppressed neuroblastoma growth, 9-ING-41 was most effective. The combination of 9-ING-41 and Camptosar was effective in suppressing tumor growth of xenografts.	[20]
9-ING-41, 9-ING-87 and irinotecan	Treatment with GSK-3 inhibitors and the chemotherapeutic drug irinotecan reduced drug resistance in a breast cancer PDX model.	[21]
AR-A014418 and gemcitabine	GSK-3 inhibitor suppressed some of the genes induced by gemcitabine that are involved in drug resistance of PDAC cells.	[22]
**Combination of GSK-3 inhibitors with other inhibitors or agonists**		
9-ING-41 and either chloroquine and bafilomycin	9-ING-41 have been examined either by itself or in combination with autophagy inhibitors chloroquine and bafilomycin on RCC lines	[23]
lithium chloride, SB216763, inhibitor IX (BIO) and NF-κB inhibitors PDTC parthenolide, or BAY 11-7082 and chemotherapeutic drugs.	Combining GSK-3, NF-κB inhibitors and certain chemotherapeutic drugs resulted in increased osteosarcoma death both in vitro and in animal xenograft studies.	[24]
AR-A014418 and Troglitazone	Treatment of prostate cancer cells with GSK-3 inhibitor and PPAR agonist suppressed NF-κB activity increased cell death.	[25]
6BIO and AR-ASO	6BIO improved the targeting of antisense oligonucleotide (ASO) inhibitor and resulted in increased inhibition of AR signaling.	[26]
AR-A014418, 5-chloro-2,4-dihydroxpyridine (CDHP) and 5FU	GSK-3β inhibitor AR-A014418 induced head and neck cancer stem cells [CD44 (high)/ESA (low)] to undergo mesenchymal-to-epithelial transition (MET) back to CD44 (high)/ESA (high) cells. Furthermore, this combined treatment induced the cells to differentiate.	[27]
**Inhibitors originally developed to target other signaling molecules which also target/inhibit GSK-3 activity**		
Tivantinib	Tivantinib was initially developed as a c-MET inhibitor but it was subsequently determined to target GSK-3α and GSK-3β in lung cancer cells.	[28]
GDC-0941	GDC-0941 is a PI3K inhibitor. It increased the sensitivity of GBM cells to radiotherapy and reduced chemoresistance to temzolomide.	[29]
AktX. Lithium chloride	AktX is an AKT inhibitor. The effects AktX and lithium chloride on brain cancer cells were determined. AktX	[30]

	suppressed AKT and increased GSK-3β expression and inhibited glioma cell proliferation.	
Zidovudine	Zidovudine is an anti-viral drug. Treatment of drug resistant pancreatic cells with zidovudine resensitized the cells to gemcitabine. Zidovudine suppressed the AKT/GSK-3/SNAIL pathway.	[31]
Doxazosin	Doxazosin is an antihypertensive drug. It was observed to inhibit PI3K/AKT signaling in GBM by upregulation of active GSK-3β and TP53. Treatment with doxazosin was associated with low neurotoxicity.	[32]
Erlotinib, SU11274, XAV939, everolimus	EGFR, c-MET, WNT, mTORC1 blocker treatments in various combinations overcame drug resistance of NSCLC cells.	[33]
miR-101, temozomide	Suppression of GSK-3β by miR-101 inhibits GSK-3β expression and restored sensitivity to temozomide in brain cancer cells.	[34]
**Nutraceuticals/Natural Products which may alter GSK-3 activity**		
Curcumin	Curcumin suppressed Syk activity which inhibited AKT and induced GSK-3 activity and inhibited B lymphoma growth.	[35]
Curcumin and Tetrahydrocurcumin	Curcumin induced GSK-3 activity and inhibited WNT/β-catenin signaling and suppressed azoxymethane-induced colon carcinogenesis.	[36]
Berberine	Berberine inhibited AKT which resulted in GSK-3 activity in melanoma cells treated with alpha melanocyte stimulating hormone (α-MSH). Berberine suppressed induction of microphthalmia-associated transcription factor (MITF) and tyrosinase activity.	[37]
Berberine and lapatinib	Combining berberine with the dual EGFR and HER receptor inhibitor lapatinib decreased lapatinib-resistance of breast cancer cells. Treatment with berberine and lapatinib induced higher levels of ROS and increased GSK-3 activity and decreased c-MYC levels.	[38]
Resveratrol	Resveratrol increased GSK-3 activity which suppressed WNT/β-catenin signaling and decreased invasion and migration in breast cancer cells.	[39]
Apocynin	The effects of apocynin and resveratrol on pancreatic cancer cells were mediated by decreased levels of phosphorylated GSK-3β and ERK1/2 present in the nucleus.	[40]
Microsclerodermin A	Microsclerodermin A inhibited NF-κB activity in PDAC. Potential involvement of GSK-3.	[41]
Caffeine	Caffeine inhibited JB6 mouse epidermal cells proliferation by suppression of AKT and activation of GSK-3.	[42]
Indirubin	Indirubin inhibited GSK-3 and cyclin dependent kinase activity in leukemia cells. Indirubin may have competed for the ATP binding sites in the kinase domains of the proteins.	[43]
Tetrandrine	Tetrandrine inhibited AKT which resulted in GSK-3 activation in colon cancer cells.	[44]
Differentiation-inducing factor-1	Differentiation-inducing factor-1 inhibited AKT and induced GSK-3 activity in colon cancer cells which resulted in apoptosis.	[45]

Dioscin	The effects of dioscin on proliferation were examined with osteosarcoma cells. Dioscin inhibited AKT activity which resulted in GSK-3 activation.	[46]
Nimbolide	Nimbolide inhibited PI3K activity in oral cancer cells which resulted in increased GSK-3 activity and inhibition of cytoprotective autophagy.	[47]
Oridonin	Oridonin increased GSK-3 expression which resulted in c-MYC degradation and growth inhibition and apoptosis in leukemia cells.	[48]
Apicidin	Apicidin resistance in HCC may result from decreased GSK-3 activity and increased WNT/β-catenin activity.	[49]
Wogonin	Wogonin inhibits cell growth and induces apoptosis by inhibiting the expression of GSK-3β in lung cancer cells.	[50]
Sulforaphane	Sulforaphane treatment resulted in induction of miR-19 and suppression of GSK-3β and increased WNT/β-catenin expression.	[51]
Butyrate	Butyrate induced ROS and miR-22/SIRT-1 pathway in hepatic cancer cells which resulted in suppression of AKT, increased PTEN and GSK-3 and apoptosis.	[52]
Ursolic acid	Treatment of ovarian carcinoma cells with ursolic acid resulted in inhibition of GSK-3 and induction of apoptosis	[53]
Gambogenic acid	Gambogenic acid stimulated GSK-3 activity and inhibited growth in GBM cells.	[54]

References:

1. Wang, J.S.; Wang, C.L.; Wen, J.F.; Wang, Y.J.; Hu, Y.B.; Ren, H.Z. Lithium inhibits proliferation of human esophageal cancer cell line Eca-109 by inducing a G2/M cell cycle arrest. *World J Gastroenterol*. **2008**, *14*, 3982-3989.
2. Miyashita, K.; Kawakami, K.; Nakada, M.; Mai, W.; Shakoori, A.; Fujisawa, H.; Hayashi, Y.; Hamada, J.; Minamoto, T. Potential therapeutic effect of glycogen synthase kinase 3b inhibition against human glioblastoma. *Clin. Cancer Res*. **2009**, 15, 887–897.
3. Acikgoz, E.; Guler, G.; Camlar, M.; Oktem, G.; Aktug, H. Glycogen synthase kinase-3 inhibition in glioblastoma multiforme cells induces apoptosis, cell cycle arrest and changing biomolecular structure. *Spectrochim. Acta A Mol. Biomol. Spectrosc*. **2019,** *209*, 150-164.
4. Kazi, A.; Xiang, S.; Yang, H.; Delitto, D.; Trevino, J.; Jiang, R.H.Y.; Ayaz, M.; Lawrence, H.R.; Kennedy, P.; Sebti, S.M. GSK3 suppression upregulates beta-catenin and c-Myc to abrogate KRas-dependent tumors. *Nature Commun*. **2018**, *9*, 5154.
5. Schrecengost, R.S.; Green, C.L.; Zhuang, Y.; Keller, S.N.; Smith, R.A.; Maines, L.W.; Smith, C.D. In Vitro and In Vivo Antitumor and Anti-Inflammatory Capabilities of the Novel GSK3 and CDK9 Inhibitor ABC1183. *J. Pharmacol. Exp. Ther*. **2009**, *365*, 107-116.
6. Shao, J.; Teng, Y.; Padia, R.; Hong, S.; Noh, H.; Xie, X.; Mumm, J.S.; Dong, Z.; Ding, H.F.; Cowell, J.; Kim, J.; Han, J.; Huang, S. COP1 and GSK3beta cooperate to promote c-Jun degradation and inhibit breast cancer cell tumorigenesis. *Neoplasia* **2013**, *15*, 1075-1085.
7. Rinnab, L.; Schutz, S.V.; Diesch, J.; Schmid, E.; Kufer, R.; Hautmann, R.E.; Spindler, K.D.; Cronauer M.V. Inhibition of glycogen synthase kinase-3 in androgen-responsive prostate cancer cell lines: are GSK inhibitors therapeutically useful? *Neoplasia* **2008**, *10*, 624-634.
8. Yeung, J.; Esposito, M.T.; Gandillet, A.; Zeisig, B.B.; Griessinger, E.; Bonnet, D.; So, C.W. beta-Catenin mediates the establishment and drug resistance of MLL leukemic stem cells. *Cancer Cell*. **2010**, *18*, 606–618.
9. Wang, Z.; Smith, K.S.; Murphy, M.; Piloto, O.; Somervaille, T.C.; Cleary, M.L. Glycogen synthase kinase 3 in MLL leukaemia maintenance and targeted therapy. *Nature* **2008**, *455*, 1205-1209.

10. Hu, S.; Ueda, M.; Stetson, L.; Ignatz-Hoover, J.; Moreton, S.; Chakrabarti, A.; Xia, Z.; Karan, G.; de Lima, M.; Agrawal, M.K.; Wald, DN. A Novel Glycogen Synthase Kinase-3 Inhibitor Optimized for Acute Myeloid Leukemia Differentiation Activity. *Mol. Cancer. Ther.* **2016**, 15, 1485-1494.
11. Zhou, Y.; Uddin, S.; Zimmerman, T.; Kang, J.A.; Ulaszek, J.; Wickrema, A. Growth control of multiple myeloma cells through inhibition of glycogen synthase kinase-3. *Leuk. Lymphoma*. **2008**, 49, 1945–1953.
12. Taylor, A.; Harker, J.A.; Chanthong, K.; Stevenson, P.G.; Zuniga, E.I.; Rudd, C.E. Glycogen Synthase Kinase 3 Inactivation Drives T-bet-Mediated Downregulation of Co-receptor PD-1 to Enhance CD8(+) Cytolytic T Cell Responses. *Immunity.* **2016**, 44, 274–286.
13. Parameswaran, R.; Ramakrishnan, P.; Moreton, S.A.; Xia, Z.; Hou, Y.; Lee, D.A.; Gupta, K.; deLima, M.; Beck, R.C.; Wald, D.N. Repression of GSK3 restores NK cell cytotoxicity in AML patients. *Nat. Commun*. **2016**, 7, 11154.
14. Sengupta, S.; Katz, S.C.; Sengupta, S.; Sampath, P. Glycogen synthase kinase 3 inhibition lowers PD-1 expression, promotes long-term survival and memory generation in antigen-specific CAR-T cells. *Cancer Lett.* **2018**, 433, 131-139.
15. Nwankwo, N.; Zhang, Z.; Wang, T.; Collins, C.; Resta, L.; Ermisch, S.; Day, J.; Decker, R.; Kornberg, L.; Nicol, S.; et al. Phase I study of enzastaurin and bevacizumab in patients with advanced cancer: safety, efficacy and pharmacokinetics. *Invest. New Drugs*. **2013**, 31, 653-660.
16. Wu, Y.Y.; Hsieh, C.T.; Chiu, Y.M.; Chou, S.C.; Kao, J.T.; Shieh, D.C.; Lee, Y.J. GSK-3 inhibitors enhance TRAIL-mediated apoptosis in human gastric adenocarcinoma cells. *PLoS ONE* **2018**, *13*, e0208094.
17. O'Flaherty, L.; Shnyder, S.D.; Cooper, P.A.; Cross, S.J.; Wakefield, J.G.; Pardo, O.E.; Seckl, M.J.; Tavare J.M. Tumor growth suppression using a combination of taxol-based therapy and GSK3 inhibition in non-small cell lung cancer. *PLoS ONE* **2019**, *14*, e0214610.
18. Rashid, M.S.; Mazur, T.; Ji, W.; Liu, S.T.; Taylor, W.R. Analysis of the role of GSK3 in the mitotic checkpoint. *Sci. Rep*. **2018**, *8*, 14259.
19. Santoro, R.; Zanotto, M.; Simionato, F.; Zecchetto, C.; Merz, V.; Cavallini, C.; Piro, G.; Sabbadini, F.; Boschi, F.; Scarpa, A.; Melisi, D. Modulating TAK1 Expression Inhibits YAP and TAZ Oncogenic Functions in Pancreatic Cancer. *Mol. Cancer Ther*. **2020**, *19*, 247-257.
20. Ugolkov, A.V.; Bondarenko, G.I.; Dubrovskyi, O.; Berbegall, A.P.; Navarro, S.; Noguera, R.; O'Halloran, T.V.; Hendrix, M.J.; Giles, F.J.; Mazar, A.P. 9-ING-41, a small-molecule glycogen synthase kinase-3 inhibitor, is active in neuroblastoma. *Anticancer Drugs*. **2018**, *29*, 717-724.
21. Ugolkov, A.; Gaisina, I.; Zhang, J.S.; Billadeau, D.D.; White, K.; Kozikowski, A.; Jain, S.; Cristofanilli, M.; Giles, F.; O'Halloran, T.; Cryns, V.L.; Mazar, A.P. GSK-3 inhibition overcomes chemoresistance in human breast cancer. *Cancer Lett*. **2016**, *380*, 384-392.
22. Shimasaki, T.; Ishigaki, Y.; Nakamura, Y.; Takata, T.; Nakaya, N.; Nakajima, H.; Sato, I.; Zhao, X.; Kitano, A.; Kawakami, K.; Tanaka, T.; Takegami, T.; Tomosugi, N.; Minamoto, T.; Motoo, Y. Glycogen synthase kinase 3beta inhibition sensitizes pancreatic cancer cells to gemcitabine. *J. Gastroenterol*. **2012**, *47*, 321-333.
23. Anraku, T.; Kuroki, H.; Kazama, A.; Bilim, V.; Tasaki, M.; Schmitt, D.; Mazar, A.; Giles, F.J.; Ugolkov, A.; Tomita, Y. Clinically relevant GSK-3beta inhibitor 9-ING-41 is active as a single agent and in combination with other antitumor therapies in human renal cancer. *Int. J. Mol. Med*. **2020**, *45*, 315-323.
24. Tang, Q.L.; Xie, X.B.; Wang, J.; Chen, Q.; Han, A.J.; Zou, C.Y.; Yin, J.Q.; Liu, D.W.; Liang, Y.; Zhao, Z.Q.; Yong, B.C.; Zhang, R.H.; Feng, Q.S.; Deng, W.G.; Zhu, X.F.; Zhou, B.P.; Zeng, Y.X.; Shen, J.N.; Kang, T. Glycogen synthase kinase-3beta, NF-kappaB signaling, and tumorigenesis of human osteosarcoma. *J. Nat. Cancer Inst.* **2012**, *104*, 749-763.
25. Ban, J.O.; Oh, J.H.; Son, S.M.; Won, D.; Song, H.S.; Han, S.B.; Moon, D.C.; Kang, K.W.; Song, M.J.; Hong, J.T. Troglitazone, a PPAR agonist, inhibits human prostate cancer cell growth through inactivation of NFkappaB via suppression of GSK-3beta expression. *Cancer Biol. Ther*. **2011**, *12*, 288-296.
26. Zhang, X.; Castanotto, D.; Nam, S.; Horne, D.;Stein, C. 6BIO enhances oligonucleotide activity in cells: a potential combinatorial anti-androgen receptor therapy in prostate cancer cells. *Mol. Ther*. **2017**, *25*, 79-91.
27. Shigeishi, H.; Biddle, A.; Gammon, L.; Rodini, C.O.; Yamasaki, M.; Seino, S.; Sugiyama, M.; Takechi, M.; Mackenzie, I.C. Elevation in 5-FU-induced apoptosis in head and neck cancer stem cells by a combination of CDHP and GSK3beta inhibitors. *J. Oral Pathol. Med*. **2015**, *44*, 201-207.

28. Remsing Rix, L.L.; Kuenzi, B.M.; Luo, Y.; Remily-Wood, E.; Kinose, F.; Wright, G.; Li, J.; Koomen, J.M.; Haura, E.B.; Lawrence, H.R.; Rix, U. GSK3 alpha and beta are new functionally relevant targets of tivantinib in lung cancer cells. *ACS Chem. Biol*. **2014**, 9, 353-358.
29. Shi, F.; Guo, H.; Zhang, R.; Liu, H.; Wu, L.; Wu, Q.; Liu, J.; Liu, T.; Zhang, Q. The PI3K inhibitor GDC-0941 enhances radiosensitization and reduces chemoresistance to temozolomide in GBM cell lines. *Neuroscience*. **2017**, 346, 298-308.
30. Atkins, R.J.; Dimou, J.; Paradiso, L.; Morokoff, A.P.; Kaye, A.H.; Drummond, K.J.; Hovens, C.M. Regulation of glycogen synthase kinase-3 beta (GSK-3β) by the Akt pathway in gliomas. *J. Clin. Neurosci.* **2012**, 19, 1558–1563.
31. Namba, T.; Kodama, R.; Moritomo, S.; Hoshino, T.; Mizushima, T. Zidovudine, an anti-viral drug, resensitizes gemcitabine-resistant pancreatic cancer cells to gemcitabine by inhibition of the Akt-GSK3beta-Snail pathway. *Cell Death Dis*. **2015**, *6*, e1795.
32. Gaelzer, M.M.; Coelho, B.P.; de Quadros, A.H.; Hoppe, J.B.; Terra, S.R.; Guerra, M.C.; Usach, V.; Guma, F.C.; Gonçalves, C.A.; Setton-Avruj, P.; et al. Phosphatidylinositol 3-Kinase/AKT Pathway Inhibition by Doxazosin Promotes Glioblastoma Cells Death, Upregulation of p53 and Triggers Low Neurotoxicity. *PLoS One*. **2016**, 11, e0154612.
33. Botting, G.M.; Rastogi, I.; Chhabra, G.; Nlend, M.; Puri, N. Mechanism of resistance and novel targets mediating resistance to EGFR and c-Met tyrosine kinase inhibitors in non-small cell lung cancer. *PLoS ONE* **2015**, *10*, e0136155.
34. Tian, T.; Mingyi, M.; Qiu, X.; Qiu, Y. MicroRNA-101 reverses temozolomide resistance by inhibition of GSK3β in glioblastoma. *Oncotarget*. **2016,** 7, 79584-79595.
35. Gururajan, M.; Dasu, T.; Shahidain, S.; Jennings, C.D.; Robertson, D.A.; Rangnekar, V.M.; Bondada, S. Spleen tyrosine kinase (Syk), a novel target of curcumin, is required for B lymphoma growth. *J. Immunol*. **2007**, *178*, 111-121.
36. Lai, C.S.; Wu, J.C.; Yu, S.F.; Badmaev, V.; Nagabhushanam, K.; Ho, C.T.; Pan, M.H. Tetrahydrocurcumin is more effective than curcumin in preventing azoxymethane-induced colon carcinogenesis. *Mol. Nutr. Food Res*. **2011**, *55*, 1819-1828.
37. Song, Y.C.; Lee, Y.; Kim, H.M.; Hyun, M.Y.; Lim, Y.Y.; Song, K.Y.; Kim, B.J. Berberine regulates melanin synthesis by activating PI3K/AKT, ERK and GSK3β in B16F10 melanocytes *Int. J. Mol. Med*. **2015**, *35*, 1011-1016.
38. Zhang, R.; Qiao, H.; Chen, S.; Chen, X.; Dou, K.; Wei, L.; Zhang, J. Berberine reverses lapatinib resistance of HER2-positive breast cancer cells by increasing the level of ROS. *Cancer Biol. Ther***.** **2016**, *17*, 925-934.
39. Tsai, J.H.; Hsu, L.S.; Lin, C.L.; Hong, H.M.; Pan, M.H.; Way, T.D.; Chen, W.J. 3,5,4'-Trimethoxystilbene, a natural methoxylated analog of resveratrol, inhibits breast cancer cell invasiveness by downregulation of PI3K/Akt and Wnt/β-catenin signaling cascades and reversal of epithelial-mesenchymal transition. *Toxicol. Appl. Pharmacol*. **2013**, *272*, 746-756.
40. Kato, A.; Naiki-Ito, A.; Nakazawa, T.; Hayashi, K.; Naitoh, I.; Miyabe, K.; Shimizu, S.; Kondo, H.; Nishi, Y.; Yoshida, M.; Umemura, S.; Hori, Y.; Mori, T.; Tsutsumi, M.; Kuno, T.; Suzuki, S.; Kato, H.; Ohara, H.; Joh, T.; Takahashi, S. Chemopreventive effect of resveratrol and apocynin on pancreatic carcinogenesis via modulation of nuclear phosphorylated GSK3β and ERK1/2. *Oncotarget* **2015**, *6*, 42963-42975.
41. Guzman, E.A.; Maers, K.; Roberts, J.; Kemami-Wangun, H.V.; Harmody, D.; Wright, A.E. The marine natural product microsclerodermin A is a novel inhibitor of the nuclear factor kappa B and induces apoptosis in pancreatic cancer cells. *Invest. New Drugs* **2015**, *33*, 86-94.
42. Hashimoto, T.; He, Z.; Ma, W.Y.; Schmid, P.C.; Bode, A.M.; Yang, C.S.; Dong, Z. Caffeine inhibits cell proliferation by G0/G1 phase arrest in JB6 cells. *Cancer Res*. **2004**, *64*, 3344-3349.
43. Wang, Y.; Hoi, P.M.; Chan, J.Y.; Lee, S.M. New perspective on the dual functions of indirubins in cancer therapy and neuroprotection. *Anticancer Agents Med. Chem*. **2014**, *14*, 1213-1219.
44. Chen, X.L.; Ren, K.H.; He, H.W.; Shao, R.G. Involvement of PI3K/AKT/GSK3beta pathway in tetrandrine-induced G1 arrest and apoptosis. *Cancer Biol. Ther***.** **2008**, *7*, 1073-1078.
45. Jingushi, K.; Nakamura, T.; Takahashi-Yanaga, F.; Matsuzaki, E.; Watanabe, Y.; Yoshihara, T.; Morimoto, S.; Sasaguri, T. Differentiation-inducing factor-1 suppresses the expression of c-Myc in the human cancer cell lines. *J. Pharm. Sci*. **2013**, *121*, 103-109.

46. Liu, W.; Zhao, Z.; Wang, Y.; Li, W.; Su, Q.; Jia, Q.; Zhang, J.; Zhang, X.; Shen, J.; Yin, J. Dioscin inhibits stem-cell-like properties and tumor growth of osteosarcoma through Akt/GSK3/beta-catenin signaling pathway. *Cell Death Dis*. **2018**, *9*, 343.
47. Sophia, J.; Kiran Kishore T.K.; Kowshik, J.; Mishra, R.; Nagini, S. Nimbolide, a neem limonoid inhibits Phosphatidyl Inositol-3 Kinase to activate Glycogen Synthase Kinase-3β in a hamster model of oral oncogenesis. *Sci. Rep*. **2016**, *6*, 22192.
48. Huang, H.L.; Weng, H.Y.; Wang, L.Q.; Yu, C.H.; Huang, Q.J.; Zhao, P.P.; Wen, J.Z.; Zhou, H.; Qu, L.H. Triggering Fbw7-mediated proteasomal degradation of c-Myc by oridonin induces cell growth inhibition and apoptosis. *Mol. Cancer Ther*. **2012**, *11*, 1155-1165.
49. Tu, C.C.; Cheng, L.H.; Hsu, H.H.; Chen, L.M.; Lin, Y.M.; Chen, M.C.; Lee, N.H.; Tsai, F.J.; Huang, C.Y.; Wu, W.J. Activation of snail and EMT-like signaling via the IKKalphabeta/NF-kappaB pathway in Apicidin-resistant HA22T hepatocellular carcinoma cells. *Chin. J. Physiol*. **2013**, *56*, 326–333.
50. Chen, X.M.; Bai, Y.; Zhong, Y.J.; Xie, X.L.; Long, H.W.; Yang, Y.Y.; Wu, S.G.; Jia, Q.; Wang, X.H. Wogonin has multiple anti-cancer effects by regulating c-Myc/SKP2/Fbw7alpha and HDAC1/HDAC2 pathways and inducing apoptosis in human lung adenocarcinoma cell line A549. *PLoS ONE* **2013**, *8*, e79201.
51. Zhu, J.; Wang, S.; Chen, Y.; Li, X.; Jiang, Y.; Yang, X.; Li, Y.; Wang, X.; Meng, Y.; Zhu, M.; Ma, X.; Huang, C.; Wu, R.; Xie, C.; Geng, S.; Wu, J.; Zhong, C.; Han, H. miR-19 targeting of GSK3β mediates sulforaphane suppression of lung cancer stem cells. *J. Nutr. Biochem*. **2017**, *44*, 80-91.
52. Pant, K.; Yadav, A.K.; Gupta, P.; Islam, R.; Saraya, A.; Venugopal, S.K. Butyrate induces ROS-mediated apoptosis by modulating miR-22/SIRT-1 pathway in hepatic cancer cells. *Redox Biol*. **2017**, *12*, 340-349.
53. Song, Y.H.; Jeong, S.J.; Kwon, H.Y.; Kim, B.; Kim, S.H.; Yoo, D.Y. Ursolic acid from Oldenlandia diffusa induces apoptosis via activation of caspases and phosphorylation of glycogen synthase kinase 3 beta in SK-OV-3 ovarian cancer cells. *Biol. Pharm. Bull*. **2012**, *35*,1022-1028.
54. Chen, H.B.; Zhou, L.Z.; Mei, L.; Shi, X.J.; Wang, X.S.; Li, Q.L.; Huang, L. Gambogenic acid-induced time- and dose-dependent growth inhibition and apoptosis involving Akt pathway inactivation in U251 glioblastoma cells. *J. Nat. Med.* **2012**, 66, 62-69.

© 2020 by the authors. Submitted for possible open access publication under the terms and conditions of the Creative Commons Attribution (CC BY) license (http://creativecommons.org/licenses/by/4.0/).
